# Sarcopenia evaluated by EASL/AASLD computed tomography-based criteria predicts mortality in patients with cirrhosis: A systematic review and meta-analysis

**DOI:** 10.1016/j.jhepr.2024.101113

**Published:** 2024-05-06

**Authors:** Elton Dajti, Susana G. Rodrigues, Federica Perazza, Luigi Colecchia, Giovanni Marasco, Matteo Renzulli, Giovanni Barbara, Francesco Azzaroli, Annalisa Berzigotti, Antonio Colecchia, Federico Ravaioli

**Affiliations:** 1 Gastroenterology Unit, IRCCS Azienda Ospedaliero-Universitaria di Bologna, Bologna, Italy; 2 Department of Medical and Surgical Sciences (DIMEC), University of Bologna, Bologna, Italy; 3 Department of Visceral Surgery and Medicine, Inselspital, Bern University Hospital, Bern, Switzerland; 4 IRCCS Azienda Ospedaliero-Universitaria di Bologna, Bologna, Italy; 5 Department of Radiology, IRCCS Azienda Ospedaliero-Universitaria di Bologna, Bologna, Italy; 6 Division of Gastroenterology, Azienda Ospedaliero-Universitaria di Modena and University of Modena and Reggio Emilia, Modena, Italy; 7 Division of Internal Medicine, Hepatobiliary and Immunoallergic Diseases, IRCCS Azienda Ospedaliero-Universitaria di Bologna, Bologna, Italy

**Keywords:** sarcopenia, malnutrition, skeletal muscle index, liver transplantation, survival, meta-analysis

## Abstract

**Background & Aims:**

Sarcopenia is associated with increased morbidity and mortality in patients with cirrhosis, but its definition in current literature is very heterogeneous. We performed a systematic review and meta-analysis to assess the association between mortality and sarcopenia evaluated by computed tomography (CT) in patients with cirrhosis, both overall and stratified for the criteria used to define sarcopenia.

**Methods:**

Medline, Embase, Scopus, and Cochrane Library were searched up to January 2023. We included studies assessing sarcopenia presence with CT scans and providing data on the risk of mortality. Adjusted hazard ratios (HRs) and 95% CIs were pooled using a random-effects model.

**Results:**

Thirty-nine studies comprising 12,827 patients were included in the meta-analysis. The summary prevalence of sarcopenia was 44% (95% CI 38-50%). The presence of sarcopenia (any definition) was an independent predictor of mortality with an adjusted HR of 2.07 (95% CI 1.81-2.36), and the result was consistent in all subgroup analyses. The prognostic role of the EASL/AASLD criteria was confirmed for the first time with an HR of 1.86 (95% CI 1.53-2.26) (n = 14 studies). The cut-offs used to define sarcopenia based on psoas muscle parameters varied among studies, thus, a subgroup analysis was not feasible. There was no substantial heterogeneity for the main estimates and no significant risk of publication bias.

**Conclusions:**

Sarcopenia on CT is associated with a 2-fold higher risk of mortality in patients with cirrhosis. The cut-offs proposed by EASL/AASLD are prognostically relevant and should be the recommended criteria used to define sarcopenia in clinical practice.

**Impact and implications:**

Sarcopenia assessed by the reference standard (computed tomography scan) is an independent predictor of mortality in patients with cirrhosis, with a 2-fold increase in the risk of death in all sensitivity analyses. This finding is particularly valid in patients from Europe and North America, and in transplant candidates. Stratifying for the parameters and cut-offs used, we confirmed for the first time the prognostic impact of the definition proposed by EASL/AASLD, supporting their use in clinical practice. Psoas muscle assessment is promising, but data are still limited and too heterogeneous to recommend its routine use at present.

## Highlights

• A meta-analysis of 12,827 subjects found that sarcopenia was present in 44% (38-50%) of patients with cirrhosis.
• Sarcopenia assessed by CT scan predicts overall survival, but its definition in the current literature is heterogenous.
• For the first time, the prognostic relevance of the EASL/AASLD criteria was confirmed (HR 1.86, 95%-CI: 1.52-2.26, I^2^<25%).
• Psoas muscle parameters may also be prognostically relevant, but the different cut-offs limit their use in clinical practice.

## Graphical abstract

**Figure:**
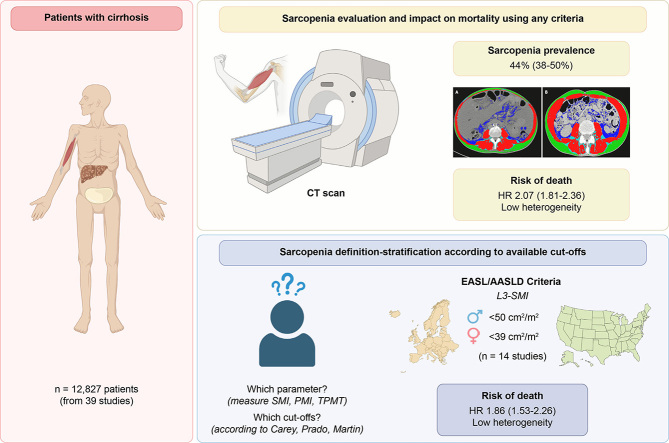

## Introduction

Sarcopenia is defined as a progressive and generalized loss of muscle mass and function and is associated with increased morbidity and mortality.^1^ Cirrhosis is a major risk factor for the development of malnutrition and sarcopenia.^2^ In addition, the presence of sarcopenia in patients with cirrhosis increases the risk of infection, falls, decompensation, and mortality.[3], [4], [5] Over the past decade, efforts to identify patients at risk, refine the definition of sarcopenia in the specific context of liver disease, and propose interventions to improve muscle dysfunction have led to the first European and North American guidelines.^6^^,^^7^

Although the prognostic relevance of sarcopenia is undisputed,^6^ many questions remain unanswered from an operational and pragmatic point of view. First, the definition of sarcopenia in liver disease usually refers to the phenotypic manifestation of loss of muscle mass, but does not consider muscle quality or function.^7^ Second, the diagnosis of sarcopenia in the current literature is very heterogeneous in terms of tests (*i.e.* cross-sectional imaging, bioimpedance analysis), measures (*i.e.* psoas muscle thickness/index, skeletal muscle index [SMI], etc.), and cut-offs used, as clearly shown in a recent systematic review.^8^

Several meta-analyses have been published on this topic.[8], [9], [10], [11] However, many large and important studies have become available since the first meta-analysis by van Vugt *et al.* in 2016.^9^ In the most recent meta-analysis by Tantai *et al.*,^8^ the impact of sarcopenia on mortality was clearly demonstrated and was consistent in all subgroup analyses. However, the study did not stratify for the specific definition (test, cut-offs) of sarcopenia, making interpretation and application of the results in clinical practice challenging.

We performed a systematic review and meta-analysis to assess the prevalence of sarcopenia and the association between mortality and sarcopenia evaluated by computed tomography (CT), both overall and stratified for the criteria used to define sarcopenia, in patients with cirrhosis.

## Material and methods

The meta-analysis was conducted and reported by the most recent MOOSE (Meta-analysis Of Observational Studies in Epidemiology) and PRISMA (Preferred Reporting Items for Systematic Reviews and Meta-Analyzes) guidelines and was registered in PROSPERO (registration number: CRD42023387648).

### Search strategy and selection criteria

We searched MEDLINE (via PubMed), Ovid Embase, Scopus, and the Cochrane Library databases up to 1^st^ of January 2023. We used the following keywords: “sarcopenia”, “muscle wasting”, “skeletal muscle”, “cirrhosis”, and “liver disease”. The full search strategy is reported in Supplementary Material 1. Further searches were performed by manual review of references. The search and selection of studies were performed by two independent investigators (ED and FR), with a third (AC) arbitrating in case of conflict regarding the eligibility of a study for inclusion. A detailed full-text assessment of potentially relevant studies was then performed. Conference abstracts and letters to the editor were also included. There were no language restrictions.

Studies were included in the review if they met the following criteria: i) sarcopenia assessed by CT scan in patients with cirrhosis; ii) available data on mortality risk according to CT measures of sarcopenia. We excluded studies if they did not provide the essential information or included patients: i) with hepatocellular carcinoma (HCC) only; ii) undergoing TIPS (transjugular intrahepatic portosystemic shunt) placement; or iii) who were candidates for liver transplantation (LT) and the outcome was post-LT mortality. The most recent or most complete publication was considered when multiple articles were found for a single study to avoid duplication. However, some studies with overlapping cohorts were allowed in subgroup analyses, if they provided prognostic information for a specific definition of sarcopenia not included in the main study.

### Data extraction and risk of bias assessment

A standardized extraction form was used to extract data from each included study. Two authors (ED and FR) extracted the following predefined data (if available): year of publication, study design, study location, number of patients, patient demographics, number of patients with viral etiology, patients with HCC, patients with decompensated cirrhosis, model for end-stage liver disease (MELD) score, main inclusion criteria (chronic liver disease, cirrhosis, acute-on-chronic liver failure [ACLF], LT candidates), definition of sarcopenia and association with mortality. If relevant data were not readily available, the authors were contacted to obtain additional data or clarification. Two authors (ED and FR) independently assessed the risk of bias using the Newcastle-Ottawa scale (NOS) for observational studies.

### Sarcopenia definition

Muscle mass was quantified as a continuous variable either as SMI (cm^2^/m^2^) at the 3^rd^ or 4^th^ lumbar vertebra (L3/L4), or as a psoas muscle (PM)-based parameter (any of transversal psoas muscle thickness [TPMT, mm] measured at the umbilical level or L3, psoas muscle area [PMA, cm^2^] or index [PMI cm^2^/m^2^]).

The main definitions of sarcopenia evaluated were:

- SMI <50 cm^2^/m^2^ in men and <39 cm^2^/m^2^ in women, as developed by Carey *et al.*^12^ in a cohort of LT candidates and endorsed by EASL and AASLD;^6^^,^^7^
- SMI <52.4 cm^2^/m^2^ in men and <38.5 cm^2^/m^2^ in women, as proposed by Prado *et al.*^13^ in a cohort of patients with solid tumors of the respiratory and gastrointestinal tract;
- SMI <53 cm^2^/m^2^ (or <43 cm^2^/m^2^ if body mass index <25 kg/m^2^) in men and <41 cm^2^/m^2^, as proposed by the same group as above in an updated paper by Martin *et al.*;^14^
- SMI <42 cm^2^/m^2^ in men and <38 cm^2^/m^2^ in women, as proposed by the Japanese Society of Hepatology (JSH).^15^

### Statistical analysis

The primary outcome was the association between the presence of sarcopenia (any definition) and mortality. Secondary outcomes were the association of sarcopenia (according to specific definitions and as a continuous value at CT measurement) with mortality.

The effect of sarcopenia on mortality was estimated by pooling in adjusted hazard ratios (HR) with 95% CIs using a random-effects model; if not available, unadjusted estimates were used.^8^^,^^9^ The variables included in the multivariate analyses of each study are summarized in Table S1. The prevalence of sarcopenia was pooled as binomial proportions with 95% CIs after Freeman-Tukey double arcsine transformation; differences between groups were tested using the random-effects meta-regression method. Heterogeneity between studies was assessed using the Q test and the I^2^ statistic; we considered an I^2^ value >50% as substantial heterogeneity.

Subgroup analyses were planned to examine the following sources of heterogeneity: region (Europe, North America, Latin America, Asia, Africa, Oceania), study design (retrospective *vs.* prospective), sample size (<150 *vs*. ≥150 patients), main etiology (viral *vs*. other), main inclusion criteria (cirrhosis *vs.* LT candidates *vs.* ACLF), inclusion of patients with HCC (yes *vs*. no), liver function (mean MELD <15 *vs*. ≥15), definition of sarcopenia (SMI- *vs.* PM-based), study quality. We also performed sensitivity analyses in which studies were pooled separately using competitive risk analysis (and reporting subhazard ratios [sHRs]). Publication bias was assessed using the funnel plot and Egger’s and Begg’s tests.

All analyses were performed with STATA version 17 (StataCorp, College Station, TX, USA) and R-Project version 4.1.1 (package meta and metafor, R Core Team 2021, Vienna, Austria).

## Results

**Fig. 1:**
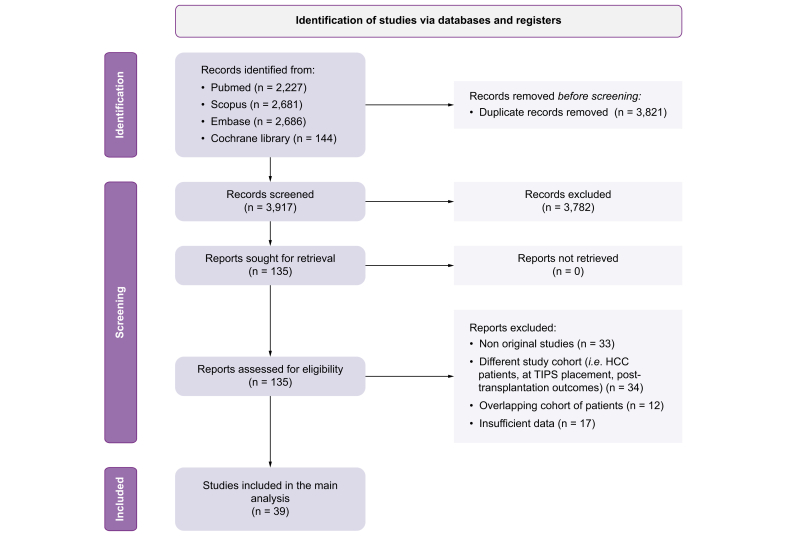
PRISMA flowchart for included studies. HCC, hepatocellular carcinoma; TIPS, transjugular portosystemic shunt.

The electronic search identified 3,917 records after removing duplicates, of which 135 were assessed for eligibility. Of these, 33 were non-original studies, 34 assessed non-target populations (*i.e.*, patients with HCC, at TIPS placement, or post-LT), 17 had insufficient data, and 12 had overlapping cohorts. Finally, 39 studies met the inclusion criteria[16], [17], [18], [19], [20], [21], [22], [23], [24], [25], [26], [27], [28], [29], [30], [31], [32], [33], [34], [35], [36], [37], [38], [39], [40], [41], [42], [43], [44], [45], [46], [47], [48], [49], [50], [51], [52], [53], [54] and were included in the main analysis, representing a total of 12,827 patients. Data from eight additional studies^12^^,^[55], [56], [57], [58], [59], [60], [61] from the overlapping cohorts were used in the subgroup analyses. Fig. 1 shows the flowchart of the selection process and details the reasons for excluding studies. Almost all studies (37/39) were rated as high quality (NOS ≥7 points) (Table 1), so no sensitivity analysis based on study quality was performed.

**Table 1:** Characteristics of included studies.

Author, Year	Country	Study type	Patients	Main etiology	HCC, n (%)	Main inclusion criteria	Child-Pugh A, n (%)	MELD score	Main sarcopenia definition	Main cut-off (M, F)∗∗	Cases, n (%)	NOS
Anand *et al.*, 2022	India	Prospective, single center	219	ArLD	0	Cirrhosis, mixed	108 (49%)	12	L3-SMI	<50, <39	170 (78%)	8
Benmassaoud *et al.*, 2022	UK	Retrospective, single center	628	Viral	176 (28%)	LT candidates	41 (7%)	14	L3-SMI	<50, <39	177 (28%)	9
Chen *et al.*, 2022	China	Retrospective, single center	223	Viral	0	Cirrhosis, mixed	79 (35%)	12	L3-SMI	Continuous	N/A	9
Dajti *et al.*, 2022	Italy	Retrospective, single center	209	Viral	40 (19%)	Cirrhosis, mixed	175 (84%)	10	L3-SMI	<50, <39	134 (64%)	7
Khan *et al.*, 2022	India	Prospective, single center	111	ArLD	0	ACLF∗	0 (0%)	28	L3-SMI	<50, <39	76 (68%)	6
Kim *et al.*, 2022	Korea	Prospective, multicenter	595	Viral	0	Cirrhosis, mixed	531 (89%)	8	L3-SMI	<50, <39	109 (18%)	8
Matsui *et al.*, 2022	Japan	Retrospective, single center	202	ArLD	0	Cirrhosis, mixed	N/A	10	L3-SMI	<42, <38	143 (71%)	8
Nardelli *et al.*, 2022	Italy	Prospective, single center	114	Viral	0	Cirrhosis, mixed	32 (28%)	13	L3-SMI	<50, <39	68 (60%)	8
Peng *et al.*, 2022	China	Retrospective, single center	433	Viral	0	ACLF	N/A	22	L3-SMI	<42, <38	250 (58%)	7
Zeng *et al.*, 2022	China	Retrospective, multicenter	480	Viral	0	Cirrhosis, mixed	112 (23%)	12	L3-SMI	<44.8, <32.5	109 (23%)	7
Cho *et al.*, 2021	Korea	Retrospective, single center	166	ArLD	0	Cirrhosis, mixed	62 (37%)	11	L3-SMI	<50, <39	79 (48%)	8
Ishizu *et al.*, 2021	Japan	Retrospective, single center	335	Viral	0	Cirrhosis, mixed	190 (57%)	N/A	L3-SMI	<42, <38	108 (32%)	8
Lai *et al.*, 2021	Italy	Retrospective, multicenter	855	Viral	424 (50%)	LT candidates	N/A	15	L3-SMI	Continuous	N/A	9
Li *et al.*, 2021	China	Retrospective, single center	171	Viral	0	ACLF	N/A	22	L3-SMI	<42, <38	95 (56%)	6
Ruiz-Margain *et al.*, 2021	Mexico, USA	Retrospective, multicenter	136	Viral	15 (11%)	Cirrhosis, mixed	46 (34%)	14	L3-SMI	<50, <39	78 (57%)	8
Sidhu *et al.*, 2021	India	Prospective, single center	161	Viral	0	LT candidates	N/A	22	L3-SMI	Continuous	N/A	8
Hou *et al.*, 2020	China	Retrospective, single center	274	Viral	0	Cirrhosis, mixed	102 (37%)	12	L3-SMI	<47, <32.5	100 (36%)	9
Kappus *et al.*, 2020	USA	Retrospective, single center	355	Viral	95 (27%)	LT candidates	N/A	19	L3-SMI	<50, <39	61 (17%)	8
Kremer *et al.*, 2020	Germany	Prospective, single center	87	ArLD	0	Cirrhosis, mixed	31 (36%)	13	L3-PMAr	<666, <424	44 (51%)	8
Mauro *et al.*, 2020	Argentina	Retrospective, single center	180	ArLD	21 (12%)	LT candidates	26 (14%)	15	L3-SMI	<50, <39	60 (33%)	8
Paternostro *et al.*, 2020	Austria	Retrospective, single center	203	ArLD	0	Cirrhosis, mixed	N/A	12	L3-TPMT	<12, <8	77 (38%)	8
Wang *et al.*, 2020	USA	Retrospective, single center	254	MASLD	0	Cirrhosis, mixed	121 (48%)	13	L4-SMI	Continuous	N/A	8
Hamaguchi *et al.*, 2019	Japan	Retrospective, single center	173	Viral	13 (8%)	LT candidates	N/A	15	L3-SMI	<40, <31	40 (23%)	8
Hanai *et al.*, 2019	Japan	Retrospective, single center	563	Viral	397 (71%)	Cirrhosis, mixed	375 (67%)	N/A	L3-SMI	<42, <38	118 (21%)	8
Lattanzi *et al.*, 2019	Italy	Retrospective, single center	249	Viral	112 (45%)	Cirrhosis, mixed	N/A	14	L3-SMI	<50, <39	109 (44%)	7
Rodrigues *et al.*, 2019	Switzerland	Retrospective, single center	84	ArLD	0	Cirrhosis, mixed	N/A	13	L3-SMI	<50, <39	50 (60%)	7
Bhanji *et al.*, 2018	Canada	Retrospective, single center	675	Viral	290 (43%)	LT candidates	105 (16%)	14	L3-SMI	<50, <39	242 (36%)	8
Ebadi *et al.*, 2018	USA, Canada	Retrospective, multicenter	353	Mixed	111 (31%)	LT candidates	N/A	16	L3-SMI	<50, <39	165 (47%)	8
Engelmann *et al.*, 2018	Germany	Retrospective, single center	795	ArLD	173 (22%)	LT candidates	112 (14%)	16	L3-SMI	Continuous	N/A	9
Gu *et al.*, 2018	Korea	Retrospective, multicenter	653	ArLD	0	Cirrhosis, mixed	N/A	11	L3-SMI	<52.4, <38.5	241 (37%)	7
Hiraoka *et al.*, 2018	Japan	Retrospective, single center	346	Viral	118 (34%)	Cirrhosis, mixed	230 (66%)	N/A	L3-PMI	<4.23, <2.5	54 (16%)	7
Huguet *et al.*, 2018	France	Retrospective, single center	173	ArLD	0	LT candidates	17 (10%)	21	U-TPMT	<15.2	57 (33%)	8
Kang *et al.*, 2018	Korea	Retrospective, single center	452	ArLD	0	LT candidates	215 (48%)	9	L3-SMI	<52.4, <38.5	190 (42%)	8
van Vugt *et al.*, 2018	Netherlands	Prospective, multicenter	585	Mixed	193 (33%)	LT candidates	N/A	14	L3-SMI	<53/<43, <41	254 (43%)	9
Nishikawa *et al.*, 2017	Japan	Retrospective, single center	206	Viral	53 (26%)	Cirrhosis, mixed	140 (68%)	N/A	L3-PMI	<6.36, <3.92	117 (57%)	8
Sinclair *et al.*, 2016	Australia	Retrospective, single center	145	Viral	33 (23%)	LT candidates	10 (7%)	18	L4-SMI	Continuous	102 (70%)	9
Wang *et al.*, 2016	USA	Prospective, single center	292	Viral	134 (46%)	LT candidates	79 (27%)	15	L3-SMI	Continuous	111 (38%)	9
Hanai *et al.*, 2015	Japan	Retrospective, single center	130	Viral	0	Cirrhosis, mixed	34 (26%)	N/A	L3-SMI	<52.4, <38.5	89 (68%)	7
Durand *et al.*, 2014	France	Retrospective, single center	562	Viral	261 (46%)	LT candidates	N/A	14	U-TPMT	Continuous	N/A	8

ACLF, acute-on-chronic liver failure; ArLD, alcohol-related liver disease; LT, liver transplant; MASLD, metabolic dysfunction-associated steatosis liver disease; N/A, not available; NOS, Newcastle-Ottawa scale; PMAr, psoas muscle area; PMI, psoas muscle index; SMI, skeletal muscle index; TPMT, transversal psoas muscle thickness.

∗ Critically ill cirrhotic patients in the intensive care unit.

∗∗ Unit for SMI and PMI: cm^2^/m^2^, TPMT: mm.

### Characteristics of the included studies

The characteristics of the included studies are shown in Table 1. Briefly, 19 studies were conducted in Asia,^16^^,^^18^^,^[20], [21], [22]^,^[24], [25], [26], [27]^,^^29^^,^^31^^,^^32^^,^^38^^,^^39^^,^^45^^,^^46^^,^^48^^,^^50^^,^^53^ 12 studies in Europe,^17^^,^^19^^,^^23^^,^^28^^,^^34^^,^^36^^,^^40^^,^^41^^,^^44^^,^^47^^,^^49^^,^^54^ six studies in North America,^30^^,^^33^^,^^37^^,^^42^^,^^43^^,^^52^ one in Latin America (Argentina)^35^ and one in Oceania (Australia).^51^ Only eight studies were prospective,^16^^,^^20^^,^^21^^,^^23^^,^^31^^,^^34^^,^^37^^,^^49^ and the rest were retrospective. The mean age of patients included varied from 43^16^ to 71 years^39^ and the proportion of male patients ranged from 40%^30^ to 100%.^51^ The proportion of patients with viral etiology ranged from 9%^49^ to 72%;^46^ 21 studies did not include patients with HCC and in the remaining studies the prevalence of HCC ranged from 8%^38^ to 71%.^39^ The main inclusion criterion was cirrhosis (both compensated and decompensated) in 21 studies, LT candidates in 15 studies, and ACLF in three studies.

### Prevalence of sarcopenia

The summary prevalence of sarcopenia was 44% (95% CI 38-50%, I^2^ = 97.2%) (Fig. S1, Table S2). In the subgroup analyses, the prevalence of sarcopenia increased progressively with increasing disease severity, according to the main inclusion criteria: 37% (95% CI 30-44%, I^2^ = 95.2%) in studies including compensated and decompensated patients with cirrhosis, 46% (95% CI 37-55%, I^2^ = 97.7%) in LT candidates and 60% (95% CI 53-66%, I^2^ not evaluable) in patients with ACLF. The summary prevalence was also lower in large studies (≥150 patients) (39%, 95% CI 33-45%, I^2^ = 59.6%) than in smaller series (<150 patients) (63%, 95% CI 57-68%, I^2^ = 97.7%). No other significant differences were found in the other subgroup and meta-regression analyses (Table S2).

### Effect of sarcopenia on mortality

From the 30 studies (n = 9,404 patients) that provided data, the presence of sarcopenia was independently associated with a 2-fold increased risk of mortality, with a summary adjusted HR of 2.07 (95% CI 1.81-2.36, I^2^ = 34.2%) (Table 2, Fig. S2). This association was robust and remained significant with a similar HR (range 1.9-2.4) in all prespecified subgroup analyses (*i.e.* study size and design, study location, main etiology, inclusion of patients with HCC, severity of liver disease, definition of sarcopenia), except in the small subgroup analysis (n = 3 studies) of patients with ACLF (summary HR 2.31, 95% CI 0.92-5.76, I^2^ = 82.4%) (Table 2).

**Table 2:** Association between sarcopenia and mortality.

Subgroup	Sarcopenia presence (dichotomized variable)			Skeletal muscle index (continuous variable)		
	Studies, n	Summary HR (95% CI)	I^2^	Studies, n	Summary HR (95% CI)	I^2^
Overall summary	30	2.07 (1.81-2.36)	34.2%	20	0.98 (0.97-0.98)	37.5%
Study type						
Retrospective	24	2.07 (1.76-2.34)	42.4%	4	0.98 (0.97-0.99)	34.5%
Prospective	6	2.04 (1.52-2.73)	0%	16	0.94 (0.90-0.97)	0%
Study size						
<150 patients	5	2.441 (1.66-3.59)	0%	3	0.97 (0.95-0.99)	0%
>150 patients	25	2.03 (1.76-2.35)	34.2%	17	0.98 (0.97-0.99)	43.1%
Region						
Asia	17	2.16 (1.76-2.64)	49.6%	7	0.97 (0.94-0.99)	57.8%
Europe	9	1.81 (1.48-2.22)	0%	5	0.99 (0.98-1)	0%
North America	3	2.01 (1.46-2.77)	16%	7	0.97 (0.96-0.98)	20.3%
Main etiology						
Viral	18	2.11 (1.73-2.57)	51.8%	14	0.98 (0.97-0.99)	45.6%
ArLD	11	2.09 (1.70-2.57)	0%	4	0.97 (0.95-0.99)	20.5%
Inclusion of HCC patients						
No	18	2.10 (1.74-2.53)	31.7%	8	0.97 (0.95-0.99)	52.8%
Yes	12	2.03 (1.66-2.48)	41%	12	0.98 (0.97-0.99)	29.2%
Main inclusion criteria						
Cirrhosis	17	2.09 (1.75-2.49)	29.4%	11	0.98 (0.97-0.99)	42%
LT candidates	11	1.99 (1.66-2.28)	9.7%	8	0.97 (0.96-0.98)	0%
ACLF	3	2.31 (0.92-5.76)	82.4%	N/A	N/A	N/A
Liver function						
Mean MELD <15	17	2.01 (1.76-2.29)	0%	10	0.98 (0.97-0.99)	11.3%
Mean MELD ≥15	8	2.30 (1.55-3.41)	58.1%	9	0.97 (0.96-0.99)	60.5%
Definition used						
SMI-based	24	2.00 (1.73-2.31)	30%	N/A	N/A	N/A
PM-based	6	2.43 (1.68-3.51)	52.8%	N/A	N/A	N/A

ACLF, acute-on-chronic liver failure; ArLD, alcohol-related liver disease; CI, confidence interval; HCC, hepatocellular carcinoma; HR, hazard ratio; LT, liver transplant; MELD, model for end-stage liver disease; N/A, not available; PM, psoas muscle; SMI, skeletal muscle index.

### Impact of sarcopenia defined by SMI on mortality

Twenty studies (n = 7,314 patients) provided data to estimate the risk of death using SMI as a continuous variable. Each 1 cm^2^/m^2^ increase in SMI was significantly associated with a reduced risk of death, with a summary adjusted HR of 0.98 (95% CI 0.97-0.98, I^2^ = 37.5%) (Fig. S3). This association remained significant in all the prespecified subgroup analyses (Table 2).

**Fig. 2:**
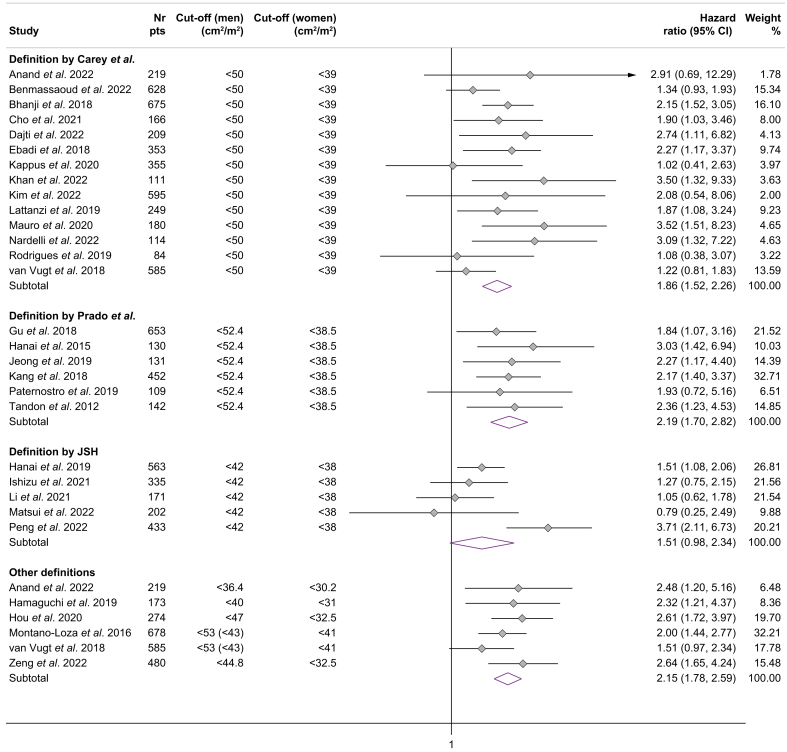
Association between mortality and sarcopenia defined according to published skeletal muscle cut-offs.

The association between mortality and the presence of sarcopenia (dichotomized) according to the most commonly used cut-offs is summarized in Fig. 2. The presence of sarcopenia defined by the EASL/AASLD criteria (Carey *et al.*^12^) increased the risk of mortality with a summary adjusted HR of 1.86 (95% CI 1.52-2.26) without substantial heterogeneity (I^2^ = 24.3%) (n = 14 studies). Similarly, sarcopenia as defined by Prado *et al.*^13^ (n = 6 studies) was independently associated with increased mortality (summary adjusted HR 2.19, 95% CI 1.70-2.82, I^2^ = 0%). In contrast, the association between the JSH criteria and mortality was not significant in a subgroup analysis of five studies (summary adjusted HR 1.51, 95% CI 0.98-2.34, I^2^ = 68.4%). The subgroup analysis for the Martin *et al.*^14^ definition of sarcopenia was not possible due to the limited number of studies (n = 2) providing data.

### Impact of sarcopenia defined by psoas muscle-based parameters on mortality

Higher values of both PMI (cm^2^/m^2^) and TPMT (mm) (continuous variables) were associated with a decreased risk of mortality, with summary adjusted HRs of 0.88 (95% CI 0.77-0.99, I^2^ = 81.4%) and 0.92 (95% CI 0.86-0.98, I^2^ = 0%), respectively (Table 3).

**Table 3:** Association between mortality and psoas muscle-based variables.

Variable	Studies, n	Summary HR (95% CI)	I^2^
PMI (continuous variable)	5	0.88 (0.77-0.99)	81.4%
TPMT (continuous variable)	4	0.92 (0.86-0.98)	0%
Psoas muscle parameter (any) (continuous variable)	10∗	0.88 (0.83-0.95)	80.8%
PMI (dichotomous variable)	6	2.32 (1.69-3.19)	55.2%
TPMT (dichotomous variable)	4	2.50 (1.48-4.23)	52.5%
Sarcopenia (any definition) (dichotomous variable)	11∗	2.29 (1.81-2.90)	43%

HR, hazard ratio; PMI, psoas muscle index; TPMT, transversal psoas muscle thickness.

∗ One study evaluated psoas muscle area.

**Fig. 3:**
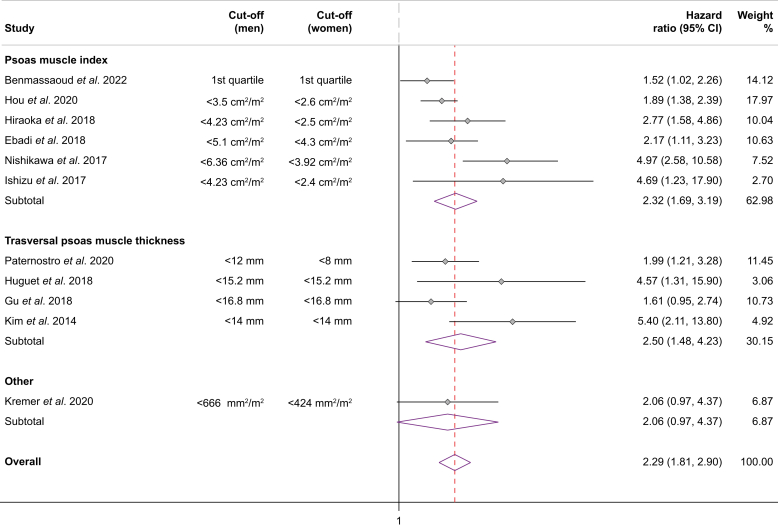
Association between mortality and sarcopenia defined according to published cut-offs based on psoas muscle.

Similarly, the presence of sarcopenia (dichotomous variable) defined by these parameters was significantly associated with an increased risk of mortality (Fig. 3). However, subgroup analysis per paired cut-offs was not possible as each study used different cut-offs to define sarcopenia.

### Sensitivity analysis and risk of bias

As HRs from Cox-proportional hazard regression may overestimate the risk of death, we planned a sensitivity analysis by separately pooling HRs and sHRs (competing-risk analysis). This analysis was only possible for the presence of sarcopenia (any definition) and sarcopenia according to the EASL/AASLD criteria, with summary adjusted sHRs of 2.05 (95% CI 1.64-2.57, I^2^ = 0%) and 2.07 (95% CI 1.47-2.93, I^2^ = 43.8%), respectively. The minimum number of studies for subgroup analysis was not reached for the other definitions of sarcopenia.

The funnel plot for the primary outcomes was symmetrical (Fig. S4). Egger’s and Begg’s tests were respectively 0.259 and 0.239, suggesting that there was no potential publication bias.

## Discussion

To our knowledge, this is the first study to systematically review and synthesize the prognostic relevance of sarcopenia according to available measures and definitions of sarcopenia on CT scans. In a meta-analysis including 39 studies and almost 13,000 patients with cirrhosis, we demonstrated that sarcopenia is an independent predictor of mortality, with an adjusted 2.1-fold increase in the risk of death. These findings were robust in all subgroup analyses, with no substantial heterogeneity. All CT-based measures of sarcopenia were independent predictors of mortality: the risk of death increased by 2.4%, 8%, and 12.4% per unit decrease in L3-SMI, TPMT, and PMI, respectively. More importantly, after stratification according to published sarcopenia cut-offs, we confirmed for the first time the prognostic relevance of the EASL/AASLD definition of sarcopenia (L3-SMI <50 cm^2^/m^2^ in men and <39 cm^2^/m^2^ in women). This approach was not possible for psoas muscle-based definitions, as all included studies used different cut-offs to define sarcopenia, so these parameters cannot currently be recommended for use in clinical practice.

A strength of this systematic review is the comprehensive search of the literature without restrictions on language, type of publication, and number of patients included. We carefully selected among studies with overlapping cohorts, pooled adjusted estimates, and performed multiple subgroup analyses to confirm the robustness of our results. The main strengths of our analysis are the inclusion of only CT-based criteria to evaluate sarcopenia, reducing the heterogeneity between studies, and stratification for the most commonly used paired cut-offs to define sarcopenia. In addition, the test for publication bias was not statistically significant, minimizing the risk of unpublished studies influencing our results.

A weakness of our findings is the heterogeneity between studies, especially for the estimates of sarcopenia prevalence (I^2^ >75%). However, the heterogeneity was not substantial (I^2^ <50%) for the main outcome and was low (I^2^ <25%) for prospective studies, studies conducted in Europe and North America, studies including LT candidates, and for the definition of sarcopenia according to EASL/AASLD criteria. Of note, the number and type of confounding factors included in multivariate analyses varied significantly among studies. This could explain in part the heterogeneity among studies, and it cannot be analyzed unless individual patient data are used.

Finally, most of the included studies were retrospective and single center, and are therefore subject to selection bias; however, the overall quality was rated as high in 95% of the studies.

To our knowledge, this is the first meta-analysis to separately analyze the different CT definitions of sarcopenia and to separately evaluate the EASL/AASLD criteria. The first meta-analysis by van Vugt *et al.*^9^ was published in 2015 and, similar to our study, only evaluated sarcopenia by CT scan. However, only six studies were included in this meta-analysis (four with overlapping cohorts) and many papers have become available since its publication. Other meta-analyses^10^^,^^11^ were limited by the inclusion of overlapping cohorts, the inclusion of post-LT patients, heterogeneous modalities for assessing sarcopenia, and high heterogeneity for the main estimates. The most recent and important meta-analysis on this topic was published by Tantai *et al.*^8^; it included only high-quality studies with at least 100 patients and showed an independent association between sarcopenia and (waiting list) mortality with an adjusted HR of 2.30 (95% CI 2.01-2.63, n = 16 studies), with no heterogeneity between studies (I^2^ = 0). However, the assessment of sarcopenia in this meta-analysis was very heterogeneous, both in terms of modality (cross-sectional imaging, bioimpedance analysis, dual-energy X-ray absorptiometry) and definitions (SMI- *vs*. PM-based cut-offs). These differences were not taken into account in their analyses, thus limiting the interpretation of the results. In this respect, the stratified analysis of parameters and paired cut-offs for the diagnosis of sarcopenia is the main novelty of our meta-analysis. Other differences between the two papers include: i) inclusion of studies assessing sarcopenia only by CT scan in our study, the gold standard for assessing sarcopenia in hepatology; ii) the exclusion of studies with <100 patients and a significant publication bias found in the paper by Tantai *et al.*; iii) exclusion of studies with an empirical proportion of patients with HCC >50%^8^; and iv) total number of patients included (12,827 patients from 39 studies *vs.* 6,965 patients from 22 studies, of whom 5,840 were evaluated with CT scan).

Sarcopenia is a common complication in patients with cirrhosis and an independent predictor of mortality. These results are particularly valid in Europe and North America and in LT candidates. More importantly, the subgroup analysis for the different definitions of sarcopenia confirmed for the first time the cut-offs proposed by Carey *et al.*^12^ and endorsed by EASL/AASLD^6^^,^^7^; the summary adjusted HR was 1.86 (95% CI 1.52-2.26) with low heterogeneity between studies. Similar results were found using the definition of sarcopenia by Prado *et al.*^13^ However, given the similarity between the cut-offs used in these two definitions (<50 *vs*. <52.4 cm^2^/m^2^ in men and <39 *vs.* <38 cm^2^/m^2^ in women), it could be argued that the EASL/AASLD criteria, derived specifically in a large cohort of patients with cirrhosis, should be the reference standard to be used in clinical practice. The JSH criteria^15^ showed a borderline association with mortality risk in our subgroup analysis, but these results should be interpreted with caution. In fact, sarcopenia is defined in Asian countries as a loss of both muscle mass and muscle function, and many studies were excluded from the analysis because they did not provide data for muscle mass separately. Finally, the assessment of the psoas muscle could be a promising and more rapid tool to assess sarcopenia, as it does not require a dedicated software to calculate. However, the cut-offs used in the current literature were different, which prevented the conduction of subgroup analyses and the recommendation of their use in routine clinical practice.

In conclusion, our data confirm that sarcopenia on CT scan is an independent predictor of mortality in patients with cirrhosis. We confirmed for the first time the prognostic relevance of the EASL/AASLD criteria for sarcopenia, but not that of the psoas muscle-based definitions, due to the different cut-offs used in the published studies; thus, the former may be the preferred criteria to assess sarcopenia in cirrhosis. Future well-designed, high-quality studies are needed to validate these results in real life and in specific contexts (*e.g.* patients with compensated cirrhosis, patients with ACLF) and geographical areas.

## Abbreviations

ACLF, acute-on-chronic liver failure; CI, confidence interval; CT, computed tomography; HCC, hepatocellular carcinoma; HR, hazard ratio; JSH, Japanese Society of Hepatology; LT, liver transplantation; MELD, model for end-stage liver disease; PMI, psoas muscle index; SMI, skeletal muscle index; TPMT, transversal psoas muscle index.

## Financial support

No grants or other financial support.

## Conflict of interest

The authors of this study declare that they do not have any conflict of interest.

Please refer to the accompanying ICMJE disclosure forms for further details.

## Authors’ contributions

ED, FR, AB, AC formulated the research questions and developed the study protocol. ED, SGR, FP, LC, GM, FR collected and extracted the data, with AB, AC, FA, GB, MR and ED providing supervision and guarantors of the study. ED, SGR, and FR analysed the data. ED, SGR, FP, LC, GM, MR, GB, FA, AB, AC, FR wrote the manuscript. All authors had full access to all the data in the study, reviewed the manuscript, and had final responsibility for the decision to submit for publication.

## Data availability statement

Data available upon reasonable request from the authors.
