# Supplementary material for: Sarcopenia evaluated by EASL/AASLD computed tomography-based criteria predicts mortality in patients with cirrhosis: A systematic review and meta-analysis
Source: JHEP Rep. 2024 May 6;6(8):101113. doi: 10.1016/j.jhepr.2024.101113 (PMC11259801; doi:10.1016/j.jhepr.2024.101113)
Supplement: Multimedia component 1 [file mmc1.pdf]

Sarcopenia evaluated by EASL/AASLD computed tomography-based criteria predicts mortality in patients with cirrhosis: A systematic review and meta-analysis

Elton Dajti, Susana G. Rodrigues, Federica Perazza, Luigi Colecchia, Giovanni Marasco, Matteo Renzulli, Giovanni Barbara, Francesco Azzaroli, Annalisa Berzigotti, Antonio Colecchia, Federico Ravaioli

Supplementary Material 1 2

Fig. S1 3

Fig. S2 4

Fig. S3 5

Fig. S4 6

Table S1. 7

Table S2 8

Supplementary Material 1 - Electronic Search strategy.

a) PUBMED (n=2227)

("liver disease*" OR "cirrhosis" OR "cirrhotic" OR ("Liver cirrhosis" [MeSH Terms])) AND ("sarcopenia" OR "sarcopenic" OR "muscle wasting" OR "muscle atrophy" OR "muscle depletion" OR "muscle mass" OR "skeletal muscle*" OR "psoas muscle*" OR ("sarcopenia" [MeSH Terms]))

b) SCOPUS (n=2681)

TITLE-ABS-KEY(("liver disease*" OR "cirrhosis") AND ("sarcopenia" OR "sarcopenic" OR "muscle wasting" OR "muscle depletion" OR "skeletal muscle*"))

c) Embase (n=2686)

'sarcopeni$' AND ('liver cirrhosis'/exp OR 'liver disease'/exp)

d) Cochrane Library (n=144)
('liver disease' OR 'cirrhosis*') AND ('sarcopenia' OR 'sarcopenic')

Fig. S1 – Summary prevalence of sarcopenia in the included studies.

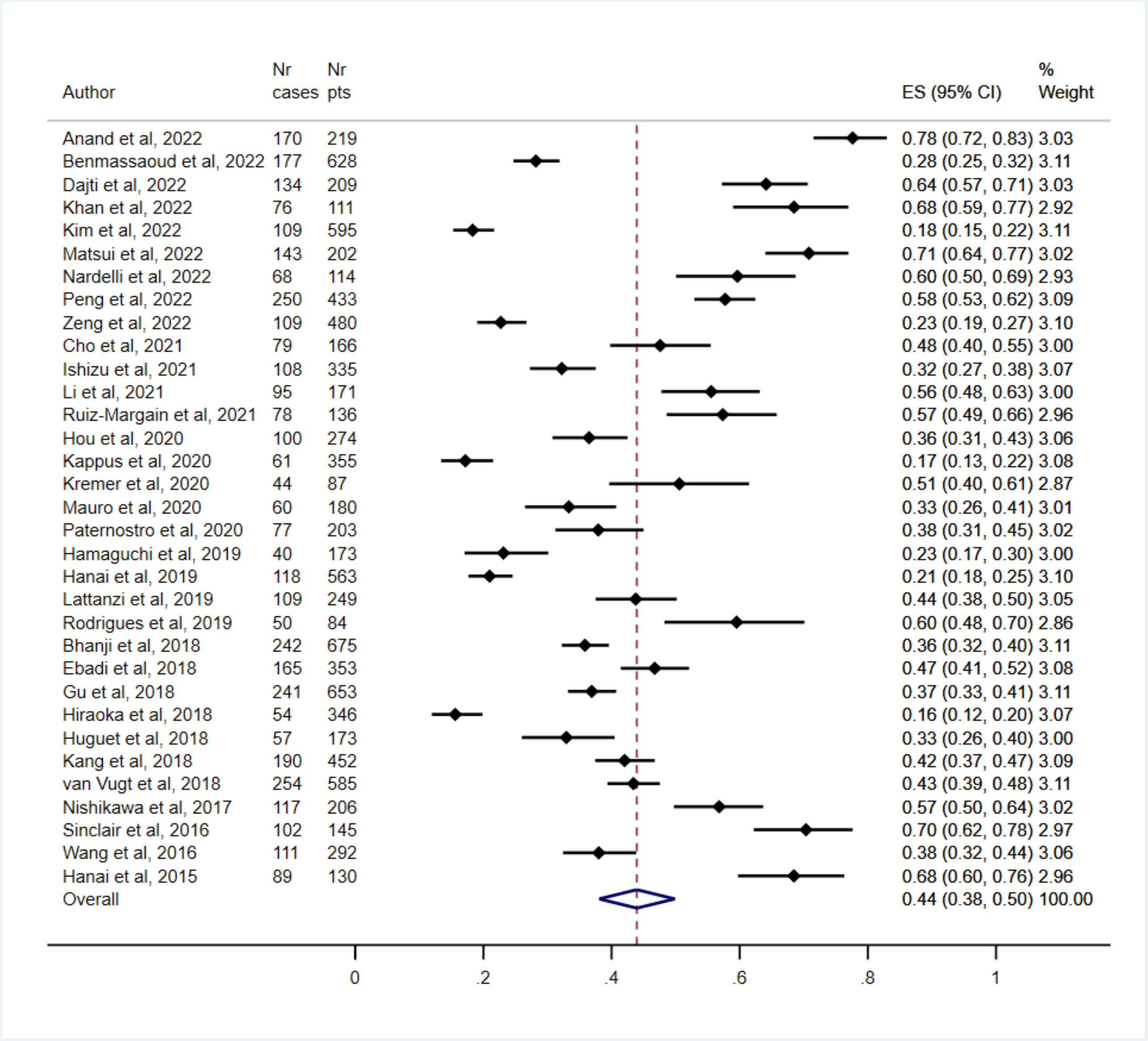

Fig. S2 – Risk of mortality in the presence of sarcopenia in cirrhotic patients.

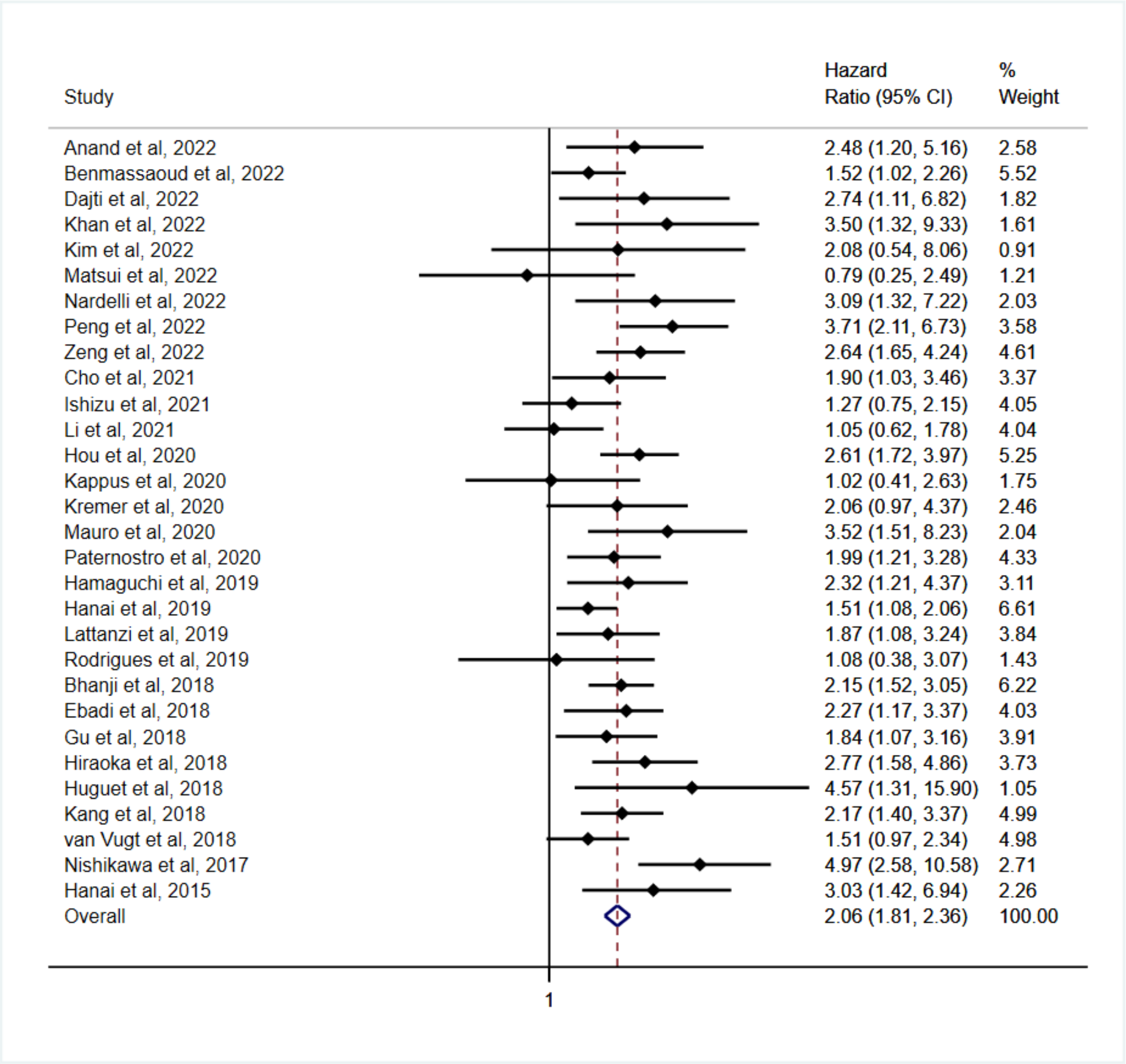

Fig. S3 – Risk of mortality with each unit increase of skeletal muscle index.

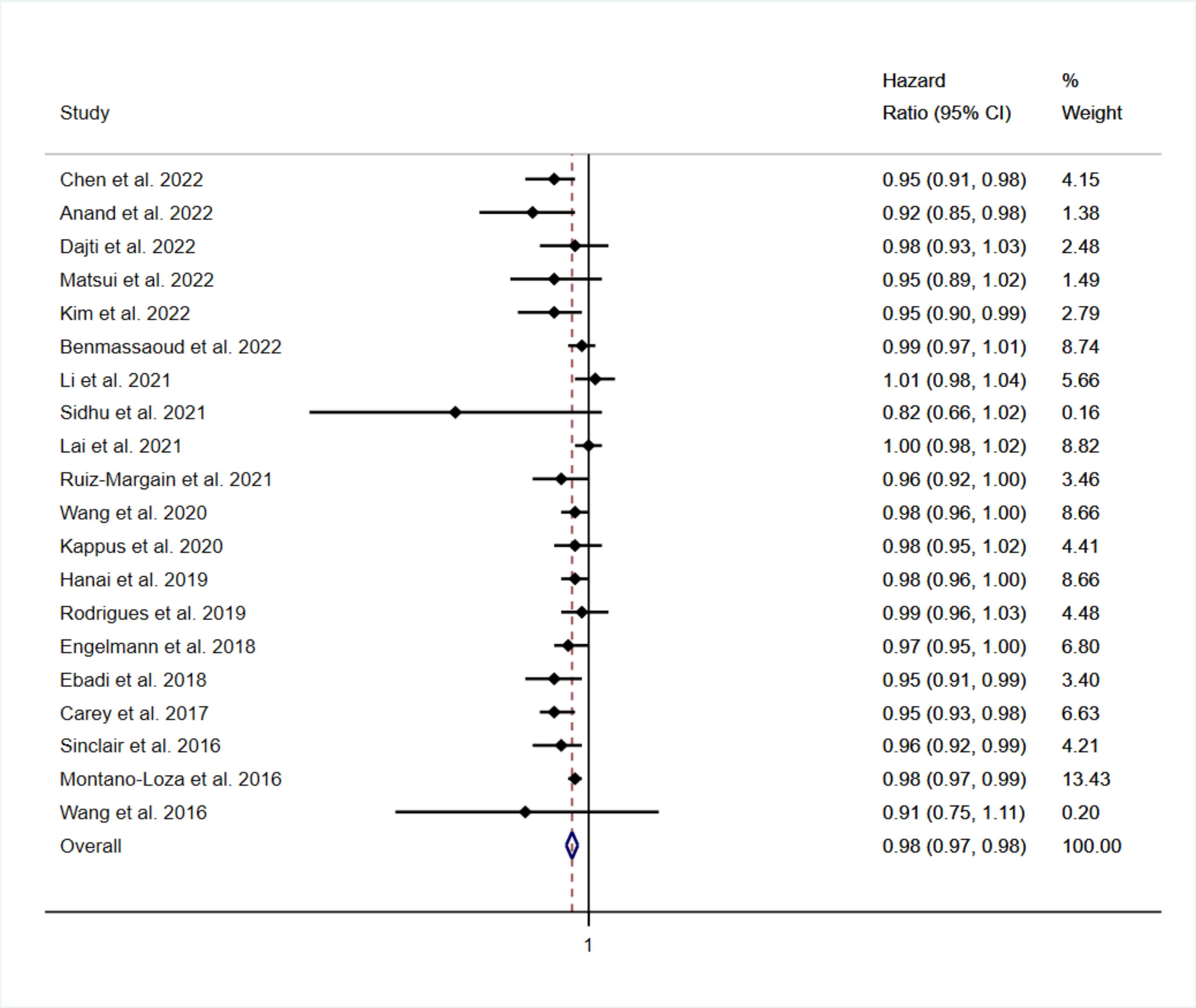

Fig. S4 - Publication bias was using the funnel plot.

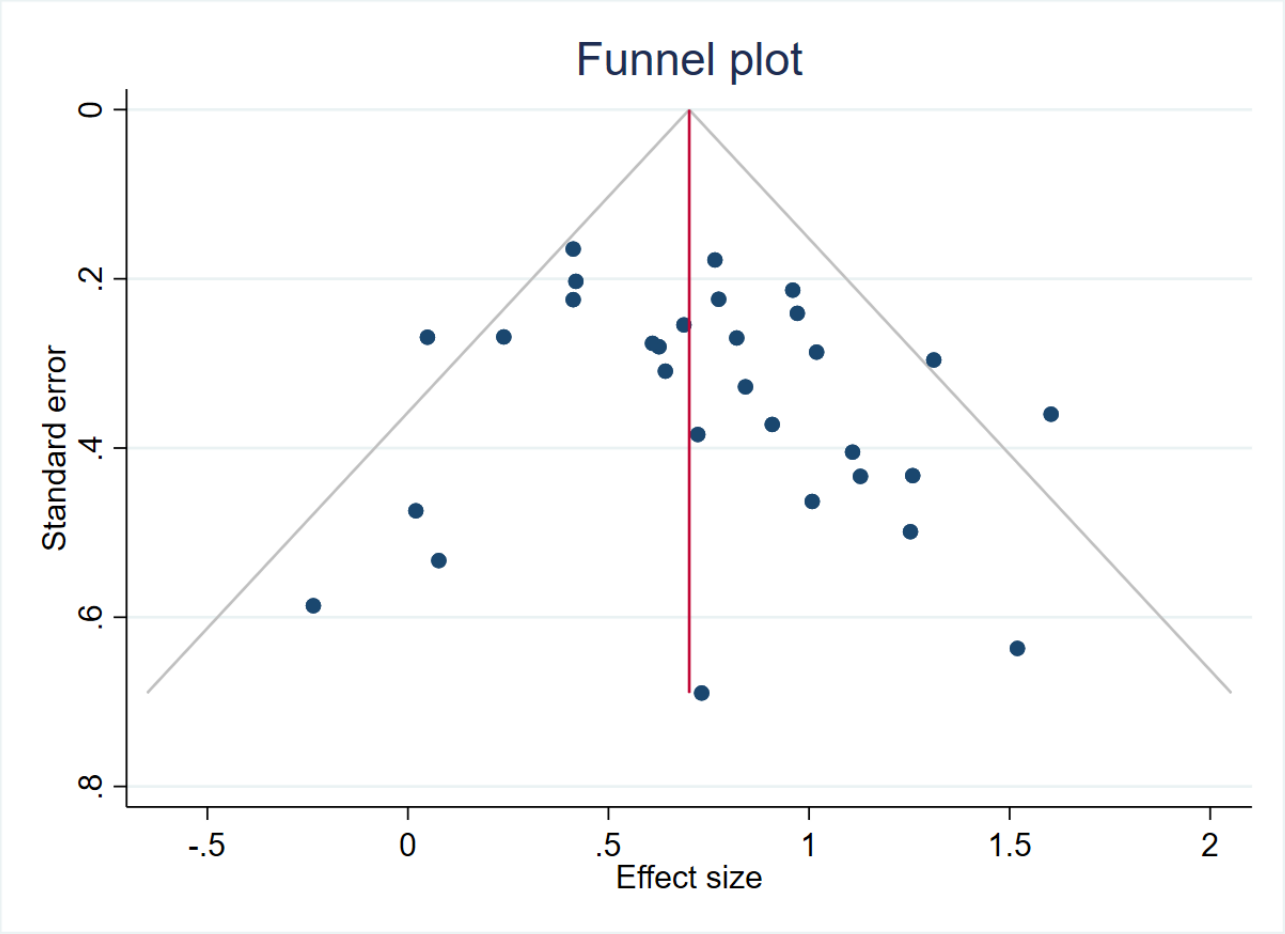

Table S1 – Variables included in the multivariate model in the evaluated studies.

Author, Year	Variables included in the multivariate analysis	Author, Year	Variables included in the multivariate
Anand et al, 2022	N/A	Paternostro et al, 2020	Age, gender, MELD, hepatic venous pressure gradient, albumin
Benmassaoud et al, 2022	N/A	Wang et al, 2020	Age, gender, Child-Pugh
Chen et al, 2022	Age, MELD, hepatic encephalopathy, infection	Hamaguchi et al, 2019	MELD, Neutrophil-to-lymphocyte ratio, myosteatosis, visceral fat
Dajti et al, 2022	Age, gender, clinically significant portal hypertension	Hanai et al, 2019	N/A
Khan et al, 2022	Sepsis, APACHE II score	Lattanzi et al, 2019	MELD, hepatic encephalopathy, myosteatosis
Kim et al, 2022	N/A	Rodrigues et al, 2019	MELD
Matsui et al, 2022	MELD, hepatic venous pressure gradient, changes in SMI	Bhanji et al, 2018	MELD, hepatic encephalopathy, Na, albumin, myosteatosis
Nardelli et al, 2022	Age, MELD, porto-systemic shunts, hepatic encephalopathy	Ebadi et al, 2018	MELD, albumin, hepatocellular carcinoma
Peng et al, 2022	Age, MELD, platelet count, alpha-fetoprotein, complications of cirrhosis	Engelmann et al, 2018	Age, gender, body mass index, MELD
Zeng et al, 2022	N/A	Gu et al, 2018	Etiology, platelet count, INR, bilirubin, albumin, creatinine, Na
Cho et al, 2021	MELD, hepatic vein pressure gradient, bone density	Hiraoka et al, 2018	Bilirubin, albumin, Child-Pugh, hepatocellular carcinoma
Ishizu et al, 2021	Age, gender, etiology, Child-Pugh	Huguet et al, 2018	N/A
Lai et al, 2021	Age, gender, body mass index, MELD, albumin	Kang et al, 2018	Gender, etiology, Child-Pugh, hepatic venous pressure gradient
Li et al, 2021	N/A	van Vugt et al, 2018	Age, MELD, hepatic encephalopathy
Ruiz-Margain et al, 2021	Age, MELD	Nishikawa et al, 2017	Age, body mass index, malnutrition, triglycerides, renal function, hepatocellular carcinoma, biomarkers
Sidhu et al, 2021	Age, gender, MELD	Sinclair et al, 2016	Age, MELD
Hou et al, 2020	Age, MELD, Child-Pugh, myosteatosis, visceral adipose tissue	Wang et al, 2016	Gender, MELD-Na, hepatocellular carcinoma, body mass index
Kappus et al, 2020	MELD	Hanai et al, 2015	Child-Pugh, use of branched chain amino acids
Kremer et al, 2020	MELD, albumin, hepatic encephalopathy, frailty	Durand et al, 2014	MELD
Mauro et al, 2020	MELD, albumin, cystatin-C		

Abbreviations APACHE: Acute Physiology and Chronic Health Evaluation MELD: Model for End-stage Liver Disease; Na: Natrium; N/A: not applicable; SMI: skeletal muscle index.

Table S2 – Prevalence of sarcopenia in subgroup analysis.

Subgroup	Nr. studies	Summary prevalence (95%-CI)	I^2	Meta-regression coefficient	p-value
**Overall**	33	44% (38-50%)	97.2%		
**Study type**				0.35 (-0.31, 0.996)	0.299
Retrospective	26	42% (36-48%)	96.9%		
Prospective	7	51% (34-67%)	98.2%		
**Study size**				-0.96 (-1.54, -0.38)	0.001
<150 pts	7	63% (57-68%)	97.7%		
>150 pts	26	39% (33-45%)	59.6%		
**Region**				-0.04 (-0.37, 0.29)	0.817
Asia	17	44% (34-53%)	98.1%		
Europe	9	46% (38-50%)	93.8%		
North America	5	38% (27-51%)	96.3%		
Other	2	50% (44-55%)	.		
**Etiology**					
Viral	20	40% (33-48%)	97.3%	-0.53 (-1.99, 0.93)	0.475
ArLD	11	51% (41-60%)	95.7%	0.94 (-0.55, 2.44)	0.217
Other	2	45% (39-52%)	.		
**Inclusion of HCC patients**				-1.32 (-2.71, 0.04)	0.056
No	18	48% (40-57%)	97.3%		
Yes	15	39% (31-47%)	97.1%		
**Main inclusion criteria**				0.25 (0.01, 0.49)	0.039
Cirrhosis	19	37% (30-44%)	95.2%		
LT candidates	11	46% (37-55%)	97.7%		
ACLF	3	60% (53-66%)	.		
**Liver function**				0.03 (-0.04, 0.09)	0.416
Mean MELD <15	18	46% (38-53%)	97.1%		
Mean MELD ≥15	10	44% (33-55%)	96.9%		
**Definition**				-0.24 (-1.002, 0.53)	0.548
Skeletal muscle-based	28	43% (36-50%)	97.5%		
Psoas muscle-based	5	38% (22-55%)	96.7%		

Abbreviations: ACLF: acute-on-chronic liver failure; ArLD: alcohol-related liver disease; CI: confidence interval; LT: liver transplant; MELD: Model for End-stage Liver Disease.

Section and Topic	Item #	Checklist item	Location where item is reported
**TITLE**			
Title	1	Identify the report as a systematic review.	1
**ABSTRACT**			
Abstract	2	See the PRISMA 2020 for Abstracts checklist.	3
**INTRODUCTION**			
Rationale	3	Describe the rationale for the review in the context of existing knowledge.	5
Objectives	4	Provide an explicit statement of the objective(s) or question(s) the review addresses.	6
**METHODS**			
Eligibility criteria	5	Specify the inclusion and exclusion criteria for the review and how studies were grouped for the syntheses.	6-7
Information sources	6	Specify all databases, registers, websites, organisations, reference lists and other sources searched or consulted to identify studies. Specify the date when each source was last searched or consulted.	6
Search strategy	7	Present the full search strategies for all databases, registers and websites, including any filters and limits used.	6
Selection process	8	Specify the methods used to decide whether a study met the inclusion criteria of the review, including how many reviewers screened each record and each report retrieved, whether they worked independently, and if applicable, details of automation tools used in the process.	6-7
Data collection process	9	Specify the methods used to collect data from reports, including how many reviewers collected data from each report, whether they worked independently, any processes for obtaining or confirming data from study investigators, and if applicable, details of automation tools used in the process.	7-8
Data items	10a	List and define all outcomes for which data were sought. Specify whether all results that were compatible with each outcome domain in each study were sought (e.g. for all measures, time points, analyses), and if not, the methods used to decide which results to collect.	7-8
	10b	List and define all other variables for which data were sought (e.g. participant and intervention characteristics, funding sources). Describe any assumptions made about any missing or unclear information.	7
Study risk of bias assessment	11	Specify the methods used to assess risk of bias in the included studies, including details of the tool(s) used, how many reviewers assessed each study and whether they worked independently, and if applicable, details of automation tools used in the process.	7
Effect measures	12	Specify for each outcome the effect measure(s) (e.g. risk ratio, mean difference) used in the synthesis or presentation of results.	8
Synthesis methods	13a	Describe the processes used to decide which studies were eligible for each synthesis (e.g. tabulating the study intervention characteristics and comparing against the planned groups for each synthesis (item #5)).	8-9
	13b	Describe any methods required to prepare the data for presentation or synthesis, such as handling of missing summary statistics, or data conversions.	8
	13c	Describe any methods used to tabulate or visually display results of individual studies and syntheses.	8-9
	13d	Describe any methods used to synthesize results and provide a rationale for the choice(s). If meta-analysis was performed, describe the model(s), method(s) to identify the presence and extent of statistical heterogeneity, and software package(s) used.	8-9
	13e	Describe any methods used to explore possible causes of heterogeneity among study results (e.g. subgroup analysis, meta-regression).	8-9
	13f	Describe any sensitivity analyses conducted to assess robustness of the synthesized results.	8-9
Reporting bias assessment	14	Describe any methods used to assess risk of bias due to missing results in a synthesis (arising from reporting biases).	9
Certainty	15	Describe any methods used to assess certainty (or confidence) in the body of evidence for an outcome.	8-9

Section and Topic	Item #	Checklist item	Location where item is reported
assessment			
**RESULTS**			
Study selection	16a	Describe the results of the search and selection process, from the number of records identified in the search to the number of studies included in the review, ideally using a flow diagram.	9
	16b	Cite studies that might appear to meet the inclusion criteria, but which were excluded, and explain why they were excluded.	9
Study characteristics	17	Cite each included study and present its characteristics.	9-10
Risk of bias in studies	18	Present assessments of risk of bias for each included study.	9
Results of individual studies	19	For all outcomes, present, for each study: (a) summary statistics for each group (where appropriate) and (b) an effect estimate and its precision (e.g. confidence/credible interval), ideally using structured tables or plots.	10-12
Results of syntheses	20a	For each synthesis, briefly summarise the characteristics and risk of bias among contributing studies.	12
	20b	Present results of all statistical syntheses conducted. If meta-analysis was done, present for each the summary estimate and its precision (e.g. confidence/credible interval) and measures of statistical heterogeneity. If comparing groups, describe the direction of the effect.	10-12
	20c	Present results of all investigations of possible causes of heterogeneity among study results.	10-12
	20d	Present results of all sensitivity analyses conducted to assess the robustness of the synthesized results.	12
Reporting biases	21	Present assessments of risk of bias due to missing results (arising from reporting biases) for each synthesis assessed.	12
Certainty of evidence	22	Present assessments of certainty (or confidence) in the body of evidence for each outcome assessed.	12
**DISCUSSION**			
Discussion	23a	Provide a general interpretation of the results in the context of other evidence.	13-14
	23b	Discuss any limitations of the evidence included in the review.	13-14
	23c	Discuss any limitations of the review processes used.	13
	23d	Discuss implications of the results for practice, policy, and future research.	15-16
**OTHER INFORMATION**			
Registration and protocol	24a	Provide registration information for the review, including register name and registration number, or state that the review was not registered.	6
	24b	Indicate where the review protocol can be accessed, or state that a protocol was not prepared.	6
	24c	Describe and explain any amendments to information provided at registration or in the protocol.	6
Support	25	Describe sources of financial or non-financial support for the review, and the role of the funders or sponsors in the review.	2
Competing interests	26	Declare any competing interests of review authors.	2
Availability of data, code and other materials	27	Report which of the following are publicly available and where they can be found: template data collection forms; data extracted from included studies; data used for all analyses; analytic code; any other materials used in the review.	2

From: Page MJ, McKenzie JE, Bossuyt PM, Boutron I, Hoffmann TC, Mulrow CD, et al. The PRISMA 2020 statement: an updated guideline for reporting systematic reviews. BMJ 2021;372:n71. doi: 10.1136/bmj.n71

MOOSE (Meta-analyses Of Observational Studies in Epidemiology) Checklist

A reporting checklist for Authors, Editors, and Reviewers of Meta-analyses of Observational Studies. You must report the page number in your manuscript where you consider each of the items listed in this checklist. If you have not included this information, either revise your manuscript accordingly before submitting or note N/A.

Reporting Criteria	Reported (Yes/No)	Reported on Page No.
**Reporting of Background**		
Problem definition	Yes	5
Hypothesis statement	Yes	6
Description of Study Outcome(s)	Yes	6
Type of exposure or intervention used	Yes	5-6
Type of study design used	Yes	5-6
Study population	Yes	5-6
**Reporting of Search Strategy**		
Qualifications of searchers (eg, librarians and investigators)	Yes	6
Search strategy, including time period included in the synthesis and keywords	Yes	6
Effort to include all available studies, including contact with authors	Yes	7
Databases and registries searched	Yes	6
Search software used, name and version, including special features used (eg, explosion)	Yes	6
Use of hand searching (eg, reference lists of obtained articles)	Yes	6
List of citations located and those excluded, including justification	Yes	6, 9
Method for addressing articles published in languages other than English	Yes	6
Method of handling abstracts and unpublished studies	Yes	6
Description of any contact with authors	Yes	7
**Reporting of Methods**		
Description of relevance or appropriateness of studies assembled for assessing the hypothesis to be tested	Yes	6
Rationale for the selection and coding of data (eg, sound clinical principles or convenience)	Yes	6
Documentation of how data were classified and coded (eg, multiple raters, blinding, and interrater reliability)	Yes	6-7
Assessment of confounding (eg, comparability of cases and controls in studies where appropriate	Yes	7-8

Reporting Criteria	Reported (Yes/No)	Reported on Page No.
Assessment of study quality, including blinding of quality assessors; stratification or regression on possible predictors of study results	Yes	7
Assessment of heterogeneity	Yes	8
Description of statistical methods (eg, complete description of fixed or random effects models, justification of whether the chosen models account for predictors of study results, dose-response models, or cumulative meta-analysis) in sufficient detail to be replicated	Yes	8-9
Provision of appropriate tables and graphics	Yes	8-9
**Reporting of Results**		
Table giving descriptive information for each study included	Yes	9
Results of sensitivity testing (eg, subgroup analysis)	Yes	10-12
Indication of statistical uncertainty of findings	Yes	10-12
**Reporting of Discussion**		
Quantitative assessment of bias (eg, publication bias)	Yes	14
Justification for exclusion (eg, exclusion of non–English-language citations)	Yes	13
Assessment of quality of included studies	Yes	14
**Reporting of Conclusions**		
Consideration of alternative explanations for observed results	Yes	14-16
Generalization of the conclusions (ie, appropriate for the data presented and within the domain of the literature review)	Yes	15-16
Guidelines for future research	Yes	15-16
Disclosure of funding source	Yes	2

Once you have completed this checklist, please save a copy and upload it as part of your submission. DO NOT include this checklist as part of the main manuscript document. It must be uploaded as a separate file.
