# Supplementary material for: Sarcopenia evaluated by EASL/AASLD computed tomography-based criteria predicts mortality in patients with cirrhosis: A systematic review and meta-analysis
Source: JHEP Rep. 2024 May 6;6(8):101113. doi: 10.1016/j.jhepr.2024.101113 (PMC11259801; doi:10.1016/j.jhepr.2024.101113)
Supplement: Multimedia component 4 [file mmc4.pdf]

Sarcopenia evaluated by EASL/AASLD computed tomography-based criteria predicts mortality in patients with cirrhosis: A systematic review and meta-analysis

Elton Dajti[1,2,*], Susana G. Rodrigues[3], Federica Perazza[2], Luigi Colecchia[2], Giovanni Marasco[2,4], Matteo Renzulli[5], Giovanni Barbara[1,2], Francesco Azzaroli[1,2], Annalisa Berzigotti[3], Antonio Colecchia[6], Federico Ravaioli[2,7]

JHEP Reports **2024**. vol. 6 | 1–9

Check for updates

Background & Aims: Sarcopenia is associated with increased morbidity and mortality in patients with cirrhosis, but its definition in current literature is very heterogeneous. We performed a systematic review and meta-analysis to assess the association between mortality and sarcopenia evaluated by computed tomography (CT) in patients with cirrhosis, both overall and stratified for the criteria used to define sarcopenia.

Methods: Medline, Embase, Scopus, and Cochrane Library were searched up to January 2023. We included studies assessing sarcopenia presence with CT scans and providing data on the risk of mortality. Adjusted hazard ratios (HRs) and 95% CIs were pooled using a random-effects model.

Results: Thirty-nine studies comprising 12,827 patients were included in the meta-analysis. The summary prevalence of sarcopenia was 44% (95% CI 38-50%). The presence of sarcopenia (any definition) was an independent predictor of mortality with an adjusted HR of 2.07 (95% CI 1.81-2.36), and the result was consistent in all subgroup analyses. The prognostic role of the EASL/AASLD criteria was confirmed for the first time with an HR of 1.86 (95% CI 1.53-2.26) (n = 14 studies). The cut-offs used to define sarcopenia based on psoas muscle parameters varied among studies, thus, a subgroup analysis was not feasible. There was no substantial heterogeneity for the main estimates and no significant risk of publication bias.

Conclusions: Sarcopenia on CT is associated with a 2-fold higher risk of mortality in patients with cirrhosis. The cut-offs proposed by EASL/AASLD are prognostically relevant and should be the recommended criteria used to define sarcopenia in clinical practice.

© 2024 The Author(s). Published by Elsevier B.V. on behalf of European Association for the Study of the Liver (EASL). This is an open access article under the CC BY license (http://creativecommons.org/licenses/by/4.0/).

Introduction

Sarcopenia is defined as a progressive and generalized loss of muscle mass and function and is associated with increased morbidity and mortality.[1] Cirrhosis is a major risk factor for the development of malnutrition and sarcopenia.[2] In addition, the presence of sarcopenia in patients with cirrhosis increases the risk of infection, falls, decompensation, and mortality.[3–5] Over the past decade, efforts to identify patients at risk, refine the definition of sarcopenia in the specific context of liver disease, and propose interventions to improve muscle dysfunction have led to the first European and North American guidelines.[6,7]

Although the prognostic relevance of sarcopenia is undisputed,[6] many questions remain unanswered from an operational and pragmatic point of view. First, the definition of sarcopenia in liver disease usually refers to the phenotypic manifestation of loss of muscle mass, but does not consider muscle quality or function.[7] Second, the diagnosis of sarcopenia in the current literature is very heterogeneous in terms of tests (*i.e.* cross-sectional imaging, bioimpedance analysis), measures (*i.e.* psoas muscle thickness/index, skeletal muscle index [SMI], etc.), and cut-offs used, as clearly shown in a recent systematic review.[8]

Several meta-analyses have been published on this topic.[8–11] However, many large and important studies have become available since the first meta-analysis by van Vugt *et al.* in 2016.[9] In the most recent meta-analysis by Tantai *et al.*,[8] the impact of sarcopenia on mortality was clearly demonstrated and was consistent in all subgroup analyses. However, the study did not stratify for the specific definition (test, cut-offs) of sarcopenia, making interpretation and application of the results in clinical practice challenging.

We performed a systematic review and meta-analysis to assess the prevalence of sarcopenia and the association between mortality and sarcopenia evaluated by computed tomography (CT), both overall and stratified for the criteria used to define sarcopenia, in patients with cirrhosis.

Material and methods

The meta-analysis was conducted and reported by the most recent MOOSE (Meta-analysis Of Observational Studies in

* Corresponding author. Address: Gastroenterology Unit, IRCCS Azienda Ospedaliero-Universitaria di Bologna, Bologna, Italy.
E-mail address: elton.dajti2@unibo.it (E. Dajti).
https://doi.org/10.1016/j.jhepr.2024.101113

Epidemiology) and PRISMA (Preferred Reporting Items for Systematic Reviews and Meta-Analyzes) guidelines and was registered in PROSPERO (registration number: CRD42023387648).

Search strategy and selection criteria

We searched MEDLINE (via PubMed), Ovid Embase, Scopus, and the Cochrane Library databases up to 1st of January 2023. We used the following keywords: "sarcopenia", "muscle wasting", "skeletal muscle", "cirrhosis", and "liver disease". The full search strategy is reported in Supplementary Material 1. Further searches were performed by manual review of references. The search and selection of studies were performed by two independent investigators (ED and FR), with a third (AC) arbitrating in case of conflict regarding the eligibility of a study for inclusion. A detailed full-text assessment of potentially relevant studies was then performed. Conference abstracts and letters to the editor were also included. There were no language restrictions.

Studies were included in the review if they met the following criteria: i) sarcopenia assessed by CT scan in patients with cirrhosis; ii) available data on mortality risk according to CT measures of sarcopenia. We excluded studies if they did not provide the essential information or included patients: i) with hepatocellular carcinoma (HCC) only; ii) undergoing TIPS (transjugular intrahepatic portosystemic shunt) placement; or iii) who were candidates for liver transplantation (LT) and the outcome was post-LT mortality. The most recent or most complete publication was considered when multiple articles were found for a single study to avoid duplication. However, some studies with overlapping cohorts were allowed in subgroup analyses, if they provided prognostic information for a specific definition of sarcopenia not included in the main study.

Data extraction and risk of bias assessment

A standardized extraction form was used to extract data from each included study. Two authors (ED and FR) extracted the following predefined data (if available): year of publication, study design, study location, number of patients, patient demographics, number of patients with viral etiology, patients with HCC, patients with decompensated cirrhosis, model for end-stage liver disease (MELD) score, main inclusion criteria (chronic liver disease, cirrhosis, acute-on-chronic liver failure [ACLF], LT candidates), definition of sarcopenia and association with mortality. If relevant data were not readily available, the authors were contacted to obtain additional data or clarification. Two authors (ED and FR) independently assessed the risk of bias using the Newcastle-Ottawa scale (NOS) for observational studies.

Sarcopenia definition

Muscle mass was quantified as a continuous variable either as SMI (cm^2/m^2) at the 3rd or 4th lumbar vertebra (L3/L4), or as a psoas muscle (PM)-based parameter (any of transversal psoas muscle thickness [TPMT, mm] measured at the umbilical level or L3, psoas muscle area [PMA, cm^2] or index [PMI cm^2/m^2]).

The main definitions of sarcopenia evaluated were:

- SMI <50 cm^2/m^2 in men and <39 cm^2/m^2 in women, as developed by Carey *et al.*[12] in a cohort of LT candidates and endorsed by EASL and AASLD;[6,7]
- SMI <52.4 cm^2/m^2 in men and <38.5 cm^2/m^2 in women, as proposed by Prado *et al.*[13] in a cohort of patients with solid tumors of the respiratory and gastrointestinal tract;
- SMI <53 cm^2/m^2 (or <43 cm^2/m^2 if body mass index <25 kg/m^2) in men and <41 cm^2/m^2, as proposed by the same group as above in an updated paper by Martin *et al.*;[14]
- SMI <42 cm^2/m^2 in men and <38 cm^2/m^2 in women, as proposed by the Japanese Society of Hepatology (JSH).[15]

Statistical analysis

The primary outcome was the association between the presence of sarcopenia (any definition) and mortality. Secondary outcomes were the association of sarcopenia (according to specific definitions and as a continuous value at CT measurement) with mortality.

The effect of sarcopenia on mortality was estimated by pooling in adjusted hazard ratios (HR) with 95% CIs using a random-effects model; if not available, unadjusted estimates were used.[8,9] The variables included in the multivariate analyses of each study are summarized in Table S1. The prevalence of sarcopenia was pooled as binomial proportions with 95% CIs after Freeman-Tukey double arcsine transformation; differences between groups were tested using the random-effects meta-regression method. Heterogeneity between studies was assessed using the Q test and the I^2 statistic; we considered an I^2 value >50% as substantial heterogeneity.

Subgroup analyses were planned to examine the following sources of heterogeneity: region (Europe, North America, Latin America, Asia, Africa, Oceania), study design (retrospective *vs.* prospective), sample size (<150 *vs.* ≥150 patients), main etiology (viral *vs.* other), main inclusion criteria (cirrhosis *vs.* LT candidates *vs.* ACLF), inclusion of patients with HCC (yes *vs.* no), liver function (mean MELD <15 *vs.* ≥15), definition of sarcopenia (SMI- *vs.* PM-based), study quality. We also performed sensitivity analyses in which studies were pooled separately using competitive risk analysis (and reporting subhazard ratios [sHRs]). Publication bias was assessed using the funnel plot and Egger's and Begg's tests.

All analyses were performed with STATA version 17 (StataCorp, College Station, TX, USA) and R-Project version 4.1.1 (package meta and metafor, R Core Team 2021, Vienna, Austria).

Results

The electronic search identified 3,917 records after removing duplicates, of which 135 were assessed for eligibility. Of these, 33 were non-original studies, 34 assessed non-target populations (*i.e.*, patients with HCC, at TIPS placement, or post-LT), 17 had insufficient data, and 12 had overlapping cohorts. Finally, 39 studies met the inclusion criteria[16–54] and were included in the main analysis, representing a total of 12,827 patients. Data from eight additional studies[12,55–61] from the overlapping cohorts were used in the subgroup analyses. Fig. 1 shows the flowchart of the selection process and details the reasons for excluding studies. Almost all studies (37/39) were rated as high quality (NOS ≥7 points) (Table 1), so no sensitivity analysis based on study quality was performed.

Characteristics of the included studies

The characteristics of the included studies are shown in Table 1. Briefly, 19 studies were conducted in

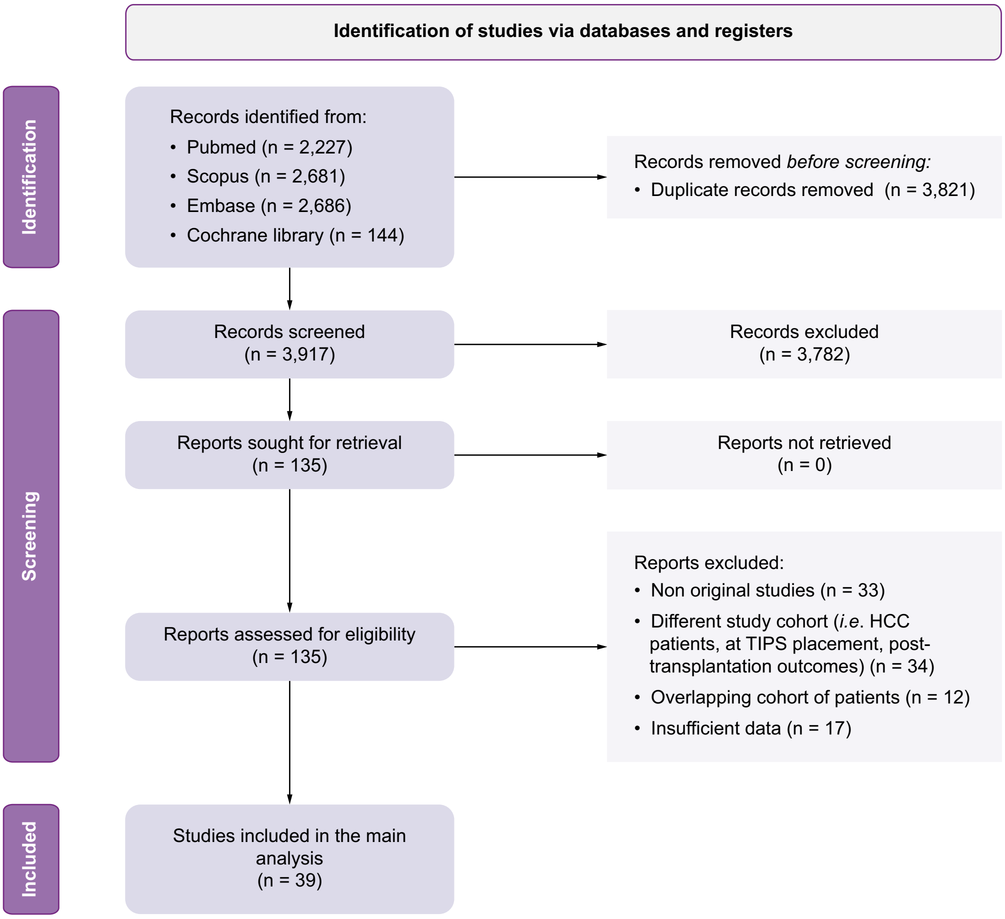

Fig. 1. PRISMA flowchart for included studies. HCC, hepatocellular carcinoma; TIPS, transjugular portosystemic shunt.

Asia,[16,18,20–22,24–27,29,31,32,38,39,45,46,48,50,53] 12 studies in Europe,[17,19,23,28,34,36,40,41,44,47,49,54] six studies in North America,[30,33,37,42,43,52] one in Latin America (Argentina)[35] and one in Oceania (Australia).[51] Only eight studies were prospective,[16,20,21,23,31,34,37,49] and the rest were retrospective. The mean age of patients included varied from 43[16] to 71 years[39] and the proportion of male patients ranged from 40%[30] to 100%.[51] The proportion of patients with viral etiology ranged from 9%[49] to 72%;[46] 21 studies did not include patients with HCC and in the remaining studies the prevalence of HCC ranged from 8%[38] to 71%.[39] The main inclusion criterion was cirrhosis (both compensated and decompensated) in 21 studies, LT candidates in 15 studies, and ACLF in three studies.

Prevalence of sarcopenia

The summary prevalence of sarcopenia was 44% (95% CI 38-50%, I^2 = 97.2%) (Fig. S1, Table S2). In the subgroup analyses, the prevalence of sarcopenia increased progressively with increasing disease severity, according to the main inclusion criteria: 37% (95% CI 30-44%, I^2 = 95.2%) in studies including compensated and decompensated patients with cirrhosis, 46% (95% CI 37-55%, I^2 = 97.7%) in LT candidates and 60% (95% CI 53-66%, I^2 not evaluable) in patients with ACLF. The summary prevalence was also lower in large studies (≥150 patients) (39%, 95% CI 33-45%, I^2 = 59.6%) than in smaller series (<150 patients) (63%, 95% CI 57-68%, I^2 = 97.7%). No other significant differences were found in the other subgroup and meta-regression analyses (Table S2).

Effect of sarcopenia on mortality

From the 30 studies (n = 9,404 patients) that provided data, the presence of sarcopenia was independently associated with a 2-fold increased risk of mortality, with a summary adjusted HR of 2.07 (95% CI 1.81-2.36, I^2 = 34.2%) (Table 2, Fig. S2). This association was robust and remained significant with a similar HR (range 1.9-2.4) in all prespecified subgroup analyses (*i.e.* study size and design, study location, main etiology, inclusion of patients with HCC, severity of liver disease, definition of sarcopenia), except in the small subgroup analysis (n = 3 studies) of patients with ACLF (summary HR 2.31, 95% CI 0.92-5.76, I^2 = 82.4%) (Table 2).

Impact of sarcopenia defined by SMI on mortality

Twenty studies (n = 7,314 patients) provided data to estimate the risk of death using SMI as a continuous variable. Each 1 cm^2/m^2 increase in SMI was significantly associated with a reduced risk of death, with a summary adjusted HR of 0.98 (95% CI 0.97-0.98, I^2 = 37.5%) (Fig. S3). This association remained significant in all the prespecified subgroup analyses (Table 2).

Table 1. Characteristics of included studies.

Author, Year	Country	Study type	Patients	Main etiology	HCC, n (%)	Main inclusion criteria	Child-Pugh A, n (%)	MELD score	Main sarcopenia definition	Main cut-off (M, F)**	Cases, n (%)	NOS
Anand *et al.*, 2022	India	Prospective, single center	219	ArLD	0	Cirrhosis, mixed	108 (49%)	12	L3-SMI	<50, <39	170 (78%)	8
Benmassaoud *et al.*, 2022	UK	Retrospective, single center	628	Viral	176 (28%)	LT candidates	41 (7%)	14	L3-SMI	<50, <39	177 (28%)	9
Chen *et al.*, 2022	China	Retrospective, single center	223	Viral	0	Cirrhosis, mixed	79 (35%)	12	L3-SMI	Continuous	N/A	9
Dajti *et al.*, 2022	Italy	Retrospective, single center	209	Viral	40 (19%)	Cirrhosis, mixed	175 (84%)	10	L3-SMI	<50, <39	134 (64%)	7
Khan *et al.*, 2022	India	Prospective, single center	111	ArLD	0	ACLF*	0 (0%)	28	L3-SMI	<50, <39	76 (68%)	6
Kim *et al.*, 2022	Korea	Prospective, multicenter	595	Viral	0	Cirrhosis, mixed	531 (89%)	8	L3-SMI	<50, <39	109 (18%)	8
Matsui *et al.*, 2022	Japan	Retrospective, single center	202	ArLD	0	Cirrhosis, mixed	N/A	10	L3-SMI	<42, <38	143 (71%)	8
Nardelli *et al.*, 2022	Italy	Prospective, single center	114	Viral	0	Cirrhosis, mixed	32 (28%)	13	L3-SMI	<50, <39	68 (60%)	8
Peng *et al.*, 2022	China	Retrospective, single center	433	Viral	0	ACLF	N/A	22	L3-SMI	<42, <38	250 (58%)	7
Zeng *et al.*, 2022	China	Retrospective, multicenter	480	Viral	0	Cirrhosis, mixed	112 (23%)	12	L3-SMI	<44.8, <32.5	109 (23%)	7
Cho *et al.*, 2021	Korea	Retrospective, single center	166	ArLD	0	Cirrhosis, mixed	62 (37%)	11	L3-SMI	<50, <39	79 (48%)	8
Ishizu *et al.*, 2021	Japan	Retrospective, single center	335	Viral	0	Cirrhosis, mixed	190 (57%)	N/A	L3-SMI	<42, <38	108 (32%)	8
Lai *et al.*, 2021	Italy	Retrospective, multicenter	855	Viral	424 (50%)	LT candidates	N/A	15	L3-SMI	Continuous	N/A	9
Li *et al.*, 2021	China	Retrospective, single center	171	Viral	0	ACLF	N/A	22	L3-SMI	<42, <38	95 (56%)	6
Ruiz-Margain *et al.*, 2021	Mexico, USA	Retrospective, multicenter	136	Viral	15 (11%)	Cirrhosis, mixed	46 (34%)	14	L3-SMI	<50, <39	78 (57%)	8
Sidhu *et al.*, 2021	India	Prospective, single center	161	Viral	0	LT candidates	N/A	22	L3-SMI	Continuous	N/A	8
Hou *et al.*, 2020	China	Retrospective, single center	274	Viral	0	Cirrhosis, mixed	102 (37%)	12	L3-SMI	<47, <32.5	100 (36%)	9
Kappus *et al.*, 2020	USA	Retrospective, single center	355	Viral	95 (27%)	LT candidates	N/A	19	L3-SMI	<50, <39	61 (17%)	8
Kremer *et al.*, 2020	Germany	Prospective, single center	87	ArLD	0	Cirrhosis, mixed	31 (36%)	13	L3-PMAr	<666, <424	44 (51%)	8
Mauro *et al.*, 2020	Argentina	Retrospective, single center	180	ArLD	21 (12%)	LT candidates	26 (14%)	15	L3-SMI	<50, <39	60 (33%)	8
Paternostro *et al.*, 2020	Austria	Retrospective, single center	203	ArLD	0	Cirrhosis, mixed	N/A	12	L3-TPMT	<12, <8	77 (38%)	8
Wang *et al.*, 2020	USA	Retrospective, single center	254	MASLD	0	Cirrhosis, mixed	121 (48%)	13	L4-SMI	Continuous	N/A	8
Hamaguchi *et al.*, 2019	Japan	Retrospective, single center	173	Viral	13 (8%)	LT candidates	N/A	15	L3-SMI	<40, <31	40 (23%)	8
Hanai *et al.*, 2019	Japan	Retrospective, single center	563	Viral	397 (71%)	Cirrhosis, mixed	375 (67%)	N/A	L3-SMI	<42, <38	118 (21%)	8
Lattanzi *et al.*, 2019	Italy	Retrospective, single center	249	Viral	112 (45%)	Cirrhosis, mixed	N/A	14	L3-SMI	<50, <39	109 (44%)	7
Rodrigues *et al.*, 2019	Switzerland	Retrospective, single center	84	ArLD	0	Cirrhosis, mixed	N/A	13	L3-SMI	<50, <39	50 (60%)	7
Bhanji *et al.*, 2018	Canada	Retrospective, single center	675	Viral	290 (43%)	LT candidates	105 (16%)	14	L3-SMI	<50, <39	242 (36%)	8
Ebadi *et al.*, 2018	USA, Canada	Retrospective, multicenter	353	Mixed	111 (31%)	LT candidates	N/A	16	L3-SMI	<50, <39	165 (47%)	8
Engelmann *et al.*, 2018	Germany	Retrospective, single center	795	ArLD	173 (22%)	LT candidates	112 (14%)	16	L3-SMI	Continuous	N/A	9
Gu *et al.*, 2018	Korea	Retrospective, multicenter	653	ArLD	0	Cirrhosis, mixed	N/A	11	L3-SMI	<52.4, <38.5	241 (37%)	7
Hiraoka *et al.*, 2018	Japan	Retrospective, single center	346	Viral	118 (34%)	Cirrhosis, mixed	230 (66%)	N/A	L3-PMI	<4.23, <2.5	54 (16%)	7
Huguet *et al.*, 2018	France	Retrospective, single center	173	ArLD	0	LT candidates	17 (10%)	21	U-TPMT	<15.2	57 (33%)	8
Kang *et al.*, 2018	Korea	Retrospective, single center	452	ArLD	0	LT candidates	215 (48%)	9	L3-SMI	<52.4, <38.5	190 (42%)	8
van Vugt *et al.*, 2018	Netherlands	Prospective, multicenter	585	Mixed	193 (33%)	LT candidates	N/A	14	L3-SMI	<53/<43, <41	254 (43%)	9
Nishikawa *et al.*, 2017	Japan	Retrospective, single center	206	Viral	53 (26%)	Cirrhosis, mixed	140 (68%)	N/A	L3-PMI	<6.36, <3.92	117 (57%)	8
Sinclair *et al.*, 2016	Australia	Retrospective, single center	145	Viral	33 (23%)	LT candidates	10 (7%)	18	L4-SMI	Continuous	102 (70%)	9
Wang *et al.*, 2016	USA	Prospective, single center	292	Viral	134 (46%)	LT candidates	79 (27%)	15	L3-SMI	Continuous	111 (38%)	9
Hanai *et al.*, 2015	Japan	Retrospective, single center	130	Viral	0	Cirrhosis, mixed	34 (26%)	N/A	L3-SMI	<52.4, <38.5	89 (68%)	7
Durand *et al.*, 2014	France	Retrospective, single center	562	Viral	261 (46%)	LT candidates	N/A	14	U-TPMT	Continuous	N/A	8

ACLF, acute-on-chronic liver failure; ArLD, alcohol-related liver disease; LT, liver transplant; MASLD, metabolic dysfunction-associated steatosis liver disease; N/A, not available; NOS, Newcastle-Ottawa scale; PMAr, psoas muscle area; PMI, psoas muscle index; SMI, skeletal muscle index; TPMT, transversal psoas muscle thickness.

*Critically ill cirrhotic patients in the intensive care unit.

**Unit for SMI and PMI: cm^2/m^2, TPMT: mm.

Table 2. Association between sarcopenia and mortality.

Subgroup	Sarcopenia presence (dichotomized variable) Studies, n	Summary HR (95% CI)	I^2	Skeletal muscle index (continuous variable) Studies, n	Summary HR (95% CI)	I^2
Overall summary	30	2.07 (1.81-2.36)	34.2%	20	0.98 (0.97-0.98)	37.5%
Study type						
Retrospective	24	2.07 (1.76-2.34)	42.4%	4	0.98 (0.97-0.99)	34.5%
Prospective	6	2.04 (1.52-2.73)	0%	16	0.94 (0.90-0.97)	0%
Study size						
<150 patients	5	2.441 (1.66-3.59)	0%	3	0.97 (0.95-0.99)	0%
>150 patients	25	2.03 (1.76-2.35)	34.2%	17	0.98 (0.97-0.99)	43.1%
Region						
Asia	17	2.16 (1.76-2.64)	49.6%	7	0.97 (0.94-0.99)	57.8%
Europe	9	1.81 (1.48-2.22)	0%	5	0.99 (0.98-1)	0%
North America	3	2.01 (1.46-2.77)	16%	7	0.97 (0.96-0.98)	20.3%
Main etiology						
Viral	18	2.11 (1.73-2.57)	51.8%	14	0.98 (0.97-0.99)	45.6%
ArLD	11	2.09 (1.70-2.57)	0%	4	0.97 (0.95-0.99)	20.5%
Inclusion of HCC patients						
No	18	2.10 (1.74-2.53)	31.7%	8	0.97 (0.95-0.99)	52.8%
Yes	12	2.03 (1.66-2.48)	41%	12	0.98 (0.97-0.99)	29.2%
Main inclusion criteria						
Cirrhosis	17	2.09 (1.75-2.49)	29.4%	11	0.98 (0.97-0.99)	42%
LT candidates	11	1.99 (1.66-2.28)	9.7%	8	0.97 (0.96-0.98)	0%
ACLF	3	2.31 (0.92-5.76)	82.4%	N/A	N/A	N/A
Liver function						
Mean MELD <15	17	2.01 (1.76-2.29)	0%	10	0.98 (0.97-0.99)	11.3%
Mean MELD ≥15	8	2.30 (1.55-3.41)	58.1%	9	0.97 (0.96-0.99)	60.5%
Definition used						
SMI-based	24	2.00 (1.73-2.31)	30%	N/A	N/A	N/A
PM-based	6	2.43 (1.68-3.51)	52.8%	N/A	N/A	N/A

ACLF, acute-on-chronic liver failure; ArLD, alcohol-related liver disease; CI, confidence interval; HCC, hepatocellular carcinoma; HR, hazard ratio; LT, liver transplant; MELD, model for end-stage liver disease; N/A, not available; PM, psoas muscle; SMI, skeletal muscle index.

The association between mortality and the presence of sarcopenia (dichotomized) according to the most commonly used cut-offs is summarized in Fig. 2. The presence of sarcopenia defined by the EASL/AASLD criteria (Carey *et al.*[12]) increased the risk of mortality with a summary adjusted HR of 1.86 (95% CI 1.52-2.26) without substantial heterogeneity (I^2 = 24.3%) (n = 14 studies). Similarly, sarcopenia as defined by Prado *et al.*[13] (n = 6 studies) was independently associated with increased mortality (summary adjusted HR 2.19, 95% CI 1.70-2.82, I^2 = 0%). In contrast, the association between the JSH criteria and mortality was not significant in a subgroup analysis of five studies (summary adjusted HR 1.51, 95% CI 0.98-2.34, I^2 = 68.4%). The subgroup analysis for the Martin *et al.*[14] definition of sarcopenia was not possible due to the limited number of studies (n = 2) providing data.

Impact of sarcopenia defined by psoas muscle-based parameters on mortality

Higher values of both PMI (cm^2/m^2) and TPMT (mm) (continuous variables) were associated with a decreased risk of mortality, with summary adjusted HRs of 0.88 (95% CI 0.77-0.99, I^2 = 81.4%) and 0.92 (95% CI 0.86-0.98, I^2 = 0%), respectively (Table 3).

Similarly, the presence of sarcopenia (dichotomous variable) defined by these parameters was significantly associated with an increased risk of mortality (Fig. 3). However, subgroup analysis per paired cut-offs was not possible as each study used different cut-offs to define sarcopenia.

Sensitivity analysis and risk of bias

As HRs from Cox-proportional hazard regression may overestimate the risk of death, we planned a sensitivity analysis by separately pooling HRs and sHRs (competing-risk analysis). This analysis was only possible for the presence of sarcopenia (any definition) and sarcopenia according to the EASL/AASLD criteria, with summary adjusted sHRs of 2.05 (95% CI 1.64-2.57, I^2 = 0%) and 2.07 (95% CI 1.47-2.93, I^2 = 43.8%), respectively. The minimum number of studies for subgroup analysis was not reached for the other definitions of sarcopenia.

The funnel plot for the primary outcomes was symmetrical (Fig. S4). Egger's and Begg's tests were respectively 0.259 and 0.239, suggesting that there was no potential publication bias.

Discussion

To our knowledge, this is the first study to systematically review and synthesize the prognostic relevance of sarcopenia according to available measures and definitions of sarcopenia on CT scans. In a meta-analysis including 39 studies and almost 13,000 patients with cirrhosis, we demonstrated that sarcopenia is an independent predictor of mortality, with an adjusted 2.1-fold increase in the risk of death. These findings were robust in all subgroup analyses, with no substantial heterogeneity. All CT-based measures of sarcopenia were independent predictors of mortality: the risk of death increased by 2.4%, 8%, and 12.4% per unit decrease in L3-SMI, TPMT, and PMI, respectively. More importantly, after stratification according to published sarcopenia cut-offs, we confirmed for the first time the prognostic relevance of the EASL/AASLD definition of sarcopenia (L3-SMI <50 cm^2/m^2 in men and <39 cm^2/m^2 in women). This approach was not possible for psoas muscle-based definitions, as all included studies used different cut-

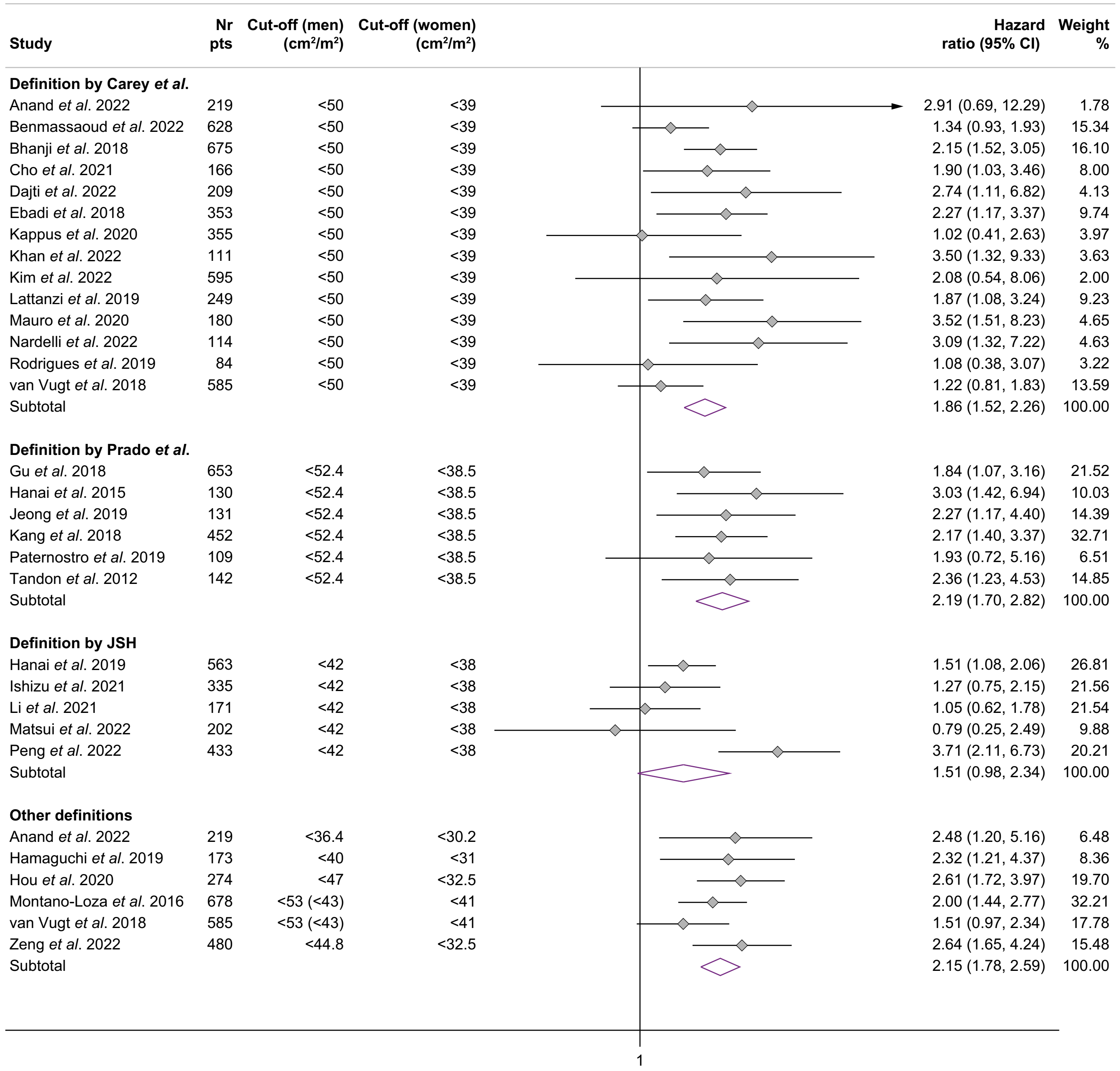

Fig. 2. Association between mortality and sarcopenia defined according to published skeletal muscle cut-offs.

offs to define sarcopenia, so these parameters cannot currently be recommended for use in clinical practice.

A strength of this systematic review is the comprehensive search of the literature without restrictions on language, type of publication, and number of patients included. We carefully selected among studies with overlapping cohorts, pooled adjusted estimates, and performed multiple subgroup analyses to confirm the robustness of our results. The main strengths of

Table 3. Association between mortality and psoas muscle-based variables.

Variable	Studies, n	Summary HR (95% CI)	I^2
PMI (continuous variable)	5	0.88 (0.77-0.99)	81.4%
TPMT (continuous variable)	4	0.92 (0.86-0.98)	0%
Psoas muscle parameter (any) (continuous variable)	10*	0.88 (0.83-0.95)	80.8%
PMI (dichotomous variable)	6	2.32 (1.69-3.19)	55.2%
TPMT (dichotomous variable)	4	2.50 (1.48-4.23)	52.5%
Sarcopenia (any definition) (dichotomous variable)	11*	2.29 (1.81-2.90)	43%

HR, hazard ratio; PMI, psoas muscle index; TPMT, transversal psoas muscle thickness.
*One study evaluated psoas muscle area.

Study	Cut-off (men)	Cut-off (women)	Hazard ratio (95% CI)	Weight %
**Psoas muscle index**				
Benmassaoud *et al.* 2022	1st quartile	1st quartile	1.52 (1.02, 2.26)	14.12
Hou *et al.* 2020	<3.5 cm²/m²	<2.6 cm²/m²	1.89 (1.38, 2.39)	17.97
Hiraoka *et al.* 2018	<4.23 cm²/m²	<2.5 cm²/m²	2.77 (1.58, 4.86)	10.04
Ebadi *et al.* 2018	<5.1 cm²/m²	<4.3 cm²/m²	2.17 (1.11, 3.23)	10.63
Nishikawa *et al.* 2017	<6.36 cm²/m²	<3.92 cm²/m²	4.97 (2.58, 10.58)	7.52
Ishizu *et al.* 2017	<4.23 cm²/m²	<2.4 cm²/m²	4.69 (1.23, 17.90)	2.70
Subtotal			2.32 (1.69, 3.19)	62.98
**Trasversal psoas muscle thickness**				
Paternostro *et al.* 2020	<12 mm	<8 mm	1.99 (1.21, 3.28)	11.45
Huguet *et al.* 2018	<15.2 mm	<15.2 mm	4.57 (1.31, 15.90)	3.06
Gu *et al.* 2018	<16.8 mm	<16.8 mm	1.61 (0.95, 2.74)	10.73
Kim *et al.* 2014	<14 mm	<14 mm	5.40 (2.11, 13.80)	4.92
Subtotal			2.50 (1.48, 4.23)	30.15
**Other**				
Kremer *et al.* 2020	<666 mm²/m²	<424 mm²/m²	2.06 (0.97, 4.37)	6.87
Subtotal			2.06 (0.97, 4.37)	6.87
**Overall**			2.29 (1.81, 2.90)	100.00

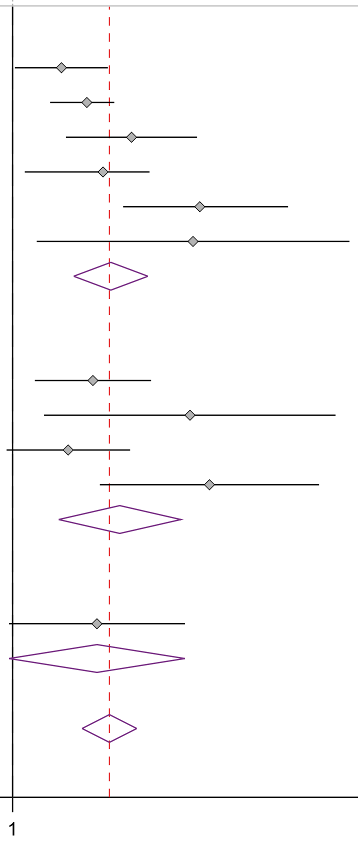

Fig. 3. Association between mortality and sarcopenia defined according to published cut-offs based on psoas muscle.

our analysis are the inclusion of only CT-based criteria to evaluate sarcopenia, reducing the heterogeneity between studies, and stratification for the most commonly used paired cut-offs to define sarcopenia. In addition, the test for publication bias was not statistically significant, minimizing the risk of unpublished studies influencing our results.

A weakness of our findings is the heterogeneity between studies, especially for the estimates of sarcopenia prevalence (I^2 >75%). However, the heterogeneity was not substantial (I^2 <50%) for the main outcome and was low (I^2 <25%) for prospective studies, studies conducted in Europe and North America, studies including LT candidates, and for the definition of sarcopenia according to EASL/AASLD criteria. Of note, the number and type of confounding factors included in multivariate analyses varied significantly among studies. This could explain in part the heterogeneity among studies, and it cannot be analyzed unless individual patient data are used.

Finally, most of the included studies were retrospective and single center, and are therefore subject to selection bias; however, the overall quality was rated as high in 95% of the studies.

To our knowledge, this is the first meta-analysis to separately analyze the different CT definitions of sarcopenia and to separately evaluate the EASL/AASLD criteria. The first meta-analysis by van Vugt *et al.*[9] was published in 2015 and, similar to our study, only evaluated sarcopenia by CT scan. However, only six studies were included in this meta-analysis (four with overlapping cohorts) and many papers have become available since its publication. Other meta-analyses[10,11] were limited by the inclusion of overlapping cohorts, the inclusion of post-LT patients, heterogeneous modalities for assessing sarcopenia, and high heterogeneity for the main estimates. The most recent and important meta-analysis on this topic was published by Tantai *et al.*[8]; it included only high-quality studies with at least 100 patients and showed an independent association between sarcopenia and (waiting list) mortality with an adjusted HR of 2.30 (95% CI 2.01-2.63, n = 16 studies), with no heterogeneity between studies (I^2 = 0). However, the assessment of sarcopenia in this meta-analysis was very heterogeneous, both in terms of modality (cross-sectional imaging, bioimpedance analysis, dual-energy X-ray absorptiometry) and definitions (SMI- *vs*. PM-based cut-offs). These differences were not taken into account in their analyses, thus limiting the interpretation of the results. In this respect, the stratified analysis of parameters and paired cut-offs for the diagnosis of sarcopenia is the main novelty of our meta-analysis. Other differences between the two papers include: i) inclusion of studies assessing sarcopenia only by CT scan in our study, the gold standard for assessing sarcopenia in hepatology; ii) the exclusion of studies with <100 patients and a significant publication bias found in the paper by Tantai *et al.*; iii) exclusion of studies with an empirical proportion of patients with HCC >50%[8]; and iv) total number of patients included

(12,827 patients from 39 studies *vs.* 6,965 patients from 22 studies, of whom 5,840 were evaluated with CT scan).

Sarcopenia is a common complication in patients with cirrhosis and an independent predictor of mortality. These results are particularly valid in Europe and North America and in LT candidates. More importantly, the subgroup analysis for the different definitions of sarcopenia confirmed for the first time the cut-offs proposed by Carey *et al.*[12] and endorsed by EASL/AASLD[6,7]; the summary adjusted HR was 1.86 (95% CI 1.52-2.26) with low heterogeneity between studies. Similar results were found using the definition of sarcopenia by Prado *et al.*[13] However, given the similarity between the cut-offs used in these two definitions (<50 *vs*. <52.4 cm^2/m^2 in men and <39 *vs.* <38 cm^2/m^2 in women), it could be argued that the EASL/AASLD criteria, derived specifically in a large cohort of patients with cirrhosis, should be the reference standard to be used in clinical practice. The JSH criteria[15] showed a borderline association with mortality risk in our subgroup analysis, but these results should be interpreted with caution. In fact, sarcopenia is defined in Asian countries as a loss of both muscle mass and muscle function, and many studies were excluded from the analysis because they did not provide data for muscle mass separately. Finally, the assessment of the psoas muscle could be a promising and more rapid tool to assess sarcopenia, as it does not require a dedicated software to calculate. However, the cut-offs used in the current literature were different, which prevented the conduction of subgroup analyses and the recommendation of their use in routine clinical practice.

In conclusion, our data confirm that sarcopenia on CT scan is an independent predictor of mortality in patients with cirrhosis. We confirmed for the first time the prognostic relevance of the EASL/AASLD criteria for sarcopenia, but not that of the psoas muscle-based definitions, due to the different cut-offs used in the published studies; thus, the former may be the preferred criteria to assess sarcopenia in cirrhosis. Future well-designed, high-quality studies are needed to validate these results in real life and in specific contexts (*e.g.* patients with compensated cirrhosis, patients with ACLF) and geographical areas.

Affiliations

[1]*Gastroenterology Unit, IRCCS Azienda Ospedaliero-Universitaria di Bologna, Bologna, Italy;* [2]*Department of Medical and Surgical Sciences (DIMEC), University of Bologna, Bologna, Italy;* [3]*Department of Visceral Surgery and Medicine, Inselspital, Bern University Hospital, Bern, Switzerland;* [4]*IRCCS Azienda Ospedaliero-Universitaria di Bologna, Bologna, Italy;* [5]*Department of Radiology, IRCCS Azienda Ospedaliero-Universitaria di Bologna, Bologna, Italy;* [6]*Division of Gastroenterology, Azienda Ospedaliero-Universitaria di Modena and University of Modena and Reggio Emilia, Modena, Italy;* [7]*Division of Internal Medicine, Hepatobiliary and Immunoallergic Diseases, IRCCS Azienda Ospedaliero-Universitaria di Bologna, Bologna, Italy*

Abbreviations

ACLF, acute-on-chronic liver failure; CI, confidence interval; CT, computed tomography; HCC, hepatocellular carcinoma; HR, hazard ratio; JSH, Japanese Society of Hepatology; LT, liver transplantation; MELD, model for end-stage liver disease; PMI, psoas muscle index; SMI, skeletal muscle index; TPMT, transversal psoas muscle index.

Financial support

No grants or other financial support.

Conflict of interest

The authors of this study declare that they do not have any conflict of interest.

Please refer to the accompanying ICMJE disclosure forms for further details.

Authors' contributions

ED, FR, AB, AC formulated the research questions and developed the study protocol. ED, SGR, FP, LC, GM, FR collected and extracted the data, with AB, AC, FA, GB, MR and ED providing supervision and guarantors of the study. ED, SGR, and FR analysed the data. ED, SGR, FP, LC, GM, MR, GB, FA, AB, AC, FR wrote the manuscript. All authors had full access to all the data in the study, reviewed the manuscript, and had final responsibility for the decision to submit for publication.

Data availability statement

Data available upon reasonable request from the authors.

Supplementary data

Supplementary data to this article can be found online at https://doi.org/10.1016/j.jhepr.2024.101113.

References

Author names in bold designate shared co-first authorship

[1] Cruz-Jentoft AJ, Bahat G, Bauer J, et al. Sarcopenia: revised European consensus on definition and diagnosis european working group on sarcopenia in older people 2 (EWGSOP2), and the extended group for EWGSOP2. Age Ageing 2019;48:16–31.

[2] Tandon P, Montano-Loza AJ, Lai JC, et al. Sarcopenia and frailty in decompensated cirrhosis. J Hepatol 2021;75:S147–S162.

[3] Tapper EB, Nikirk S, Parikh ND, et al. Falls are common, morbid, and predictable in patients with cirrhosis. J Hepatol 2021;75(3):582.

[4] Marasco G, Dajti E, Ravaioli F, et al. Clinical impact of sarcopenia assessment in patients with liver cirrhosis. Expert Rev Gastroenterol Hepatol 2021;15(4):377–388.

[5] Ge J, Kim WR, Lai JC, et al. Beyond MELD" - emerging strategies and technologies for improving mortality prediction, organ allocation and outcomes in liver transplantation. J Hepatol 2022;76(6):1318–1329.

[6] Merli M, Berzigotti A, Zelber-Sagi S, et al. EASL Clinical Practice Guidelines on nutrition in chronic liver disease. J Hepatol 2019;70(1):172–193.

[7] Lai JC, Tandon P, Bernal W, et al. Malnutrition, frailty, and sarcopenia in patients with cirrhosis: 2021 practice guidance by the American association for the study of liver diseases. Hepatology 2021;74(3):1611–1644.

[8] Tantai X, Liu Y, Yeo YH, et al. Effect of sarcopenia on survival in patients with cirrhosis: a meta-analysis. J Hepatol 2022;76(3):588–599.

[9] van Vugt JLA, Levolger S, de Bruin RWF, et al. Systematic review and meta-analysis of the impact of computed tomography–assessed skeletal muscle mass on outcome in patients awaiting or undergoing liver transplantation. Am J Transpl 2016;16(8):2277–2292.

[10] Kim G, Kang SH, Kim MY, et al. Prognostic value of sarcopenia in patients with liver cirrhosis: a systematic review and meta-analysis. PLoS One 2017;12(10).

[11] Chang KV, Chen J De, Wu WT, et al. Association of loss of muscle mass with mortality in liver cirrhosis without or before liver transplantation: a systematic review and meta-analysis. Medicine (Baltimore) 2019;98(9).

[12] Carey EJ, Lai JC, Wang CW, et al. A multicenter study to define sarcopenia in patients with end-stage liver disease. Liver Transpl 2017;23(5):625–633.

[13] Prado CM, Lieffers JR, McCargar LJ, et al. Prevalence and clinical implications of sarcopenic obesity in patients with solid tumours of the respiratory and gastrointestinal tracts: a population-based study. Lancet Oncol 2008;9(7):629–635.

[14] Martin L, Birdsell L, MacDonald N, et al. Cancer cachexia in the age of obesity: skeletal muscle depletion is a powerful prognostic factor, independent of body mass index. J Clin Oncol 2013;31(12):1539–1547.

[15] Nishikawa H, Shiraki M, Hiramatsu A, et al. Japan Society of Hepatology guidelines for sarcopenia in liver disease (1st edition): recommendation from

the working group for creation of sarcopenia assessment criteria. Hepatol Res 2016;46(10):951–963.
[16] Anand A, Mohta S, Agarwal S, et al. European working group on sarcopenia in older people (EWGSOP2) criteria with population-based skeletal muscle index best predicts mortality in asians with cirrhosis. J Clin Exp Hepatol 2022;12(1):52–60.
[17] Benmassaoud A, Roccarina D, Arico FM, et al. Sex is a major effect modifier between body composition and mortality in patients with cirrhosis assessed for liver transplantation. Liver Int 2023;43(1):160–169.
[18] Chen C, Chu Y, Sheng Q, et al. Skeletal muscle index and muscle attenuation with liver cirrhosis as survival prognosticators by sex. Asia Pac J Clin Nutr 2022;31(1):24–32.
[19] **Dajti E**, **Renzulli M**, **Ravaioli F**, et al. The interplay between sarcopenia and portal hypertension predicts ascites and mortality in cirrhosis: sarcopenia predicts ascites and death in cirrhotic patients. Dig Liver Dis 2023;55(5):637–643.
[20] **Khan S**, **Benjamin J**, Maiwall R, et al. Sarcopenia is the independent predictor of mortality in critically ill patients with cirrhosis. J Clin Transl Res 2022;8(3):200.
[21] Kim TH, Jung YK, Yim HJ, et al. Impacts of muscle mass dynamics on prognosis of outpatients with cirrhosis. Clin Mol Hepatol 2022;28(4):876–889.
[22] Matsui T, Nagai H, Watanabe G, et al. Measurement of skeletal muscle volume is useful for predicting prognosis in patients with liver cirrhosis. Eur J Gastroenterol Hepatol 2022;34(11):1151–1157.
[23] Nardelli S, Riggio O, Gioia S, et al. Risk factors for hepatic encephalopathy and mortality in cirrhosis: the role of cognitive impairment, muscle alterations and shunts: predictors of HE. Dig Liver Dis 2022;54(8):1060–1065.
[24] Peng H, Zhang Q, Luo L, et al. A prognostic model of acute-on-chronic liver failure based on sarcopenia. Hepatol Int 2022;16(4):964–972.
[25] Zeng X, Shi ZW, Yu JJ, et al. Sarcopenia as a prognostic predictor of liver cirrhosis: a multicentre study in China. J Cachexia Sarcopenia Muscle 2021;12(6):1948–1958.
[26] Cho YS, Lee HY, Jeong JY, et al. Computed tomography-determined body composition abnormalities usefully predict long-term mortality in patients with liver cirrhosis. J Comput Assist Tomogr 2021;45(5):684–690.
[27] Ishizu Y, Ishigami M, Honda T, et al. Impact of visceral fat accumulation on the prognosis of patients with cirrhosis. Clin Nutr ESPEN 2021;42:354–360.
[28] Lai Q, Magistri P, Lionetti R, et al. Sarco-Model: a score to predict the dropout risk in the perspective of organ allocation in patients awaiting liver transplantation. Liver Int 2021;41(7):1629–1640.
[29] Li T, Xu M, Kong M, et al. Use of skeletal muscle index as a predictor of short-term mortality in patients with acute-on-chronic liver failure. Sci Rep 2021;11(1).
[30] Ruiz-Margáin A, Xie JJ, Román-Calleja BM, et al. Phase angle from bioelectrical impedance for the assessment of sarcopenia in cirrhosis with or without ascites. Clin Gastroenterol Hepatol 2021;19(9):1941–1949.e2.
[31] Sidhu SS, Saggar K, Goyal O, et al. Muscle strength and physical performance, rather than muscle mass, correlate with mortality in end-stage liver disease. Eur J Gastroenterol Hepatol 2021;33(4):555–564.
[32] Hou L, Deng Y, Fan X, et al. A sex-stratified prognostic nomogram incorporating body compositions for long-term mortality in cirrhosis. JPEN J Parenter Enteral Nutr 2021;45(2):403–413.
[33] Kappus MR, Wegermann K, Bozdogan E, et al. Use of skeletal muscle index as a predictor of wait-list mortality in patients with end-stage liver disease. Liver Transpl 2020;26(9):1090–1099.
[34] Kremer WM, Nagel M, Reuter M, et al. Validation of the clinical frailty scale for the prediction of mortality in patients with liver cirrhosis. Clin Transl Gastroenterol 2020;11(7).
[35] Mauro E, Crespo G, Martinez-Garmendia A, et al. Cystatin C and sarcopenia predict acute on chronic liver failure development and mortality in patients on the liver transplant waiting list. Transplantation 2020;11(7):e002.
[36] Paternostro R, Bardach C, Hofer BS, et al. Prognostic impact of sarcopenia in cirrhotic patients stratified by different severity of portal hypertension. Liver Int 2021;41(4):799–809.
[37] Wang NC, Zhang P, Tapper EB, et al. Automated measurements of muscle mass using deep learning can predict clinical outcomes in patients with liver disease. Am J Gastroenterol 2020;115(8):1210–1216.
[38] Hamaguchi Y, Kaido T, Okumura S, et al. Including body composition in MELD scores improves mortality prediction among patients awaiting liver transplantation. Clin Nutr 2020;39(6):1885–1892.
[39] Hanai T, Shiraki M, Imai K, et al. Reduced handgrip strength is predictive of poor survival among patients with liver cirrhosis: a sex-stratified analysis. Hepatol Res 2019;49(12):1414–1426.
[40] Lattanzi B, Nardelli S, Pigliacelli A, et al. The additive value of sarcopenia, myosteatosis and hepatic encephalopathy in the predictivity of model for end-stage liver disease. Dig Liver Dis 2019;51(11):1508–1512.
[41] Rodrigues SG, Brabandt B, Stirnimann G, et al. Adipopenia correlates with higher portal pressure in patients with cirrhosis. Liver Int 2019;39(9):1672–1681.
[42] Bhanji RA, Moctezuma-Velazquez C, Duarte-Rojo A, et al. Myosteatosis and sarcopenia are associated with hepatic encephalopathy in patients with cirrhosis. Hepatol Int 2018;12(4):377–386.
[43] Ebadi M, Wang CW, Lai JC, et al. Poor performance of psoas muscle index for identification of patients with higher waitlist mortality risk in cirrhosis. J Cachexia Sarcopenia Muscle 2018;9(6):1053–1062.
[44] Engelmann C, Schob S, Nonnenmacher I, et al. Loss of paraspinal muscle mass is a gender-specific consequence of cirrhosis that predicts complications and death. Aliment Pharmacol Ther 2018;48(11–12):1271–1281.
[45] **Gu DH**, **Kim MY**, Seo YS, et al. Clinical usefulness of psoas muscle thickness for the diagnosis of sarcopenia in patients with liver cirrhosis. Clin Mol Hepatol 2018;24(3):319–330.
[46] Hiraoka A, Kitahata S, Izumoto H, et al. Muscle volume loss a prognostic factor for death in liver cirrhosis patients and special relationship to portal hypertension. Hepatol Res 2018;48(3):E354–E359.
[47] Huguet A, Latournerie M, Debry PH, et al. The psoas muscle transversal diameter predicts mortality in patients with cirrhosis on a waiting list for liver transplantation: a retrospective cohort study. Nutrition 2018;51–52:73–79.
[48] **Kang SH**, **Jeong WK**, Baik SK, et al. Impact of sarcopenia on prognostic value of cirrhosis: going beyond the hepatic venous pressure gradient and MELD score. J Cachexia Sarcopenia Muscle 2018;9(5):860–870.
[49] van Vugt JLA, Alferink LJM, Buettner S, et al. A model including sarcopenia surpasses the MELD score in predicting waiting list mortality in cirrhotic liver transplant candidates: a competing risk analysis in a national cohort. J Hepatol 2018;68(4):707–714.
[50] Nishikawa H, Enomoto H, Ishii A, et al. Prognostic significance of low skeletal muscle mass compared with protein-energy malnutrition in liver cirrhosis. Hepatol Res 2017;47(10):1042–1052.
[51] Sinclair M, Grossmann M, Angus PW, et al. Low testosterone as a better predictor of mortality than sarcopenia in men with advanced liver disease. J Gastroenterol Hepatol 2016;31(3):661–667.
[52] Wang CW, Feng S, Covinsky KE, et al. A comparison of muscle function, mass, and quality in liver transplant candidates: results from the functional assessment in liver transplantation study. Transplantation 2016;100(8): 1692–1698.
[53] Hanai T, Shiraki M, Nishimura K, et al. Sarcopenia impairs prognosis of patients with liver cirrhosis. Nutrition 2015;31(1):193–199.
[54] Durand F, Buyse S, Francoz C, et al. Prognostic value of muscle atrophy in cirrhosis using psoas muscle thickness on computed tomography. J Hepatol 2014;60(6):1151–1157.
[55] Hou L, Deng Y, Wu H, et al. Low psoas muscle index associates with long-term mortality in cirrhosis: construction of a nomogram. Ann Transl Med 2020;8(6):358. 358.
[56] Paternostro R, Lampichler K, Bardach C, et al. The value of different CT-based methods for diagnosing low muscle mass and predicting mortality in patients with cirrhosis. Liver Int 2019;39(12):2374–2385.
[57] Jeong JY, Lim S, Sohn JH, et al. Presence of sarcopenia and its rate of change are independently associated with long-term mortality in patients with liver cirrhosis. J Korean Med Sci 2018;33(50).
[58] Ishizu Y, Ishigami M, Kuzuya T, et al. Low skeletal muscle mass predicts early mortality in cirrhotic patients with acute variceal bleeding. Nutrition 2017;42:87–91.
[59] Montano-Loza AJ, Angulo P, Meza-Junco J, et al. Sarcopenic obesity and myosteatosis are associated with higher mortality in patients with cirrhosis. J Cachexia Sarcopenia Muscle 2016;7(2):126–135.
[60] Kim TY, Kim MY, Sohn JH, et al. Sarcopenia as a useful predictor for long-term mortality in cirrhotic patients with ascites. J Korean Med Sci 2014;29(9):1253–1259.
[61] Tandon P, Ney M, Irwin I, et al. Severe muscle depletion in patients on the liver transplant wait list: its prevalence and independent prognostic value. Liver Transpl 2012;18(10):1209–1216.

Keywords: sarcopenia; malnutrition; skeletal muscle index; liver transplantation; survival; meta-analysis.
Received 2 February 2024; received in revised form 3 April 2024; accepted 24 April 2024; Available online 6 May 2024

Journal of Hepatology, Volume *6*

Supplemental information

Sarcopenia evaluated by EASL/AASLD computed tomography-based criteria predicts mortality in patients with cirrhosis: A systematic review and meta-analysis

Elton Dajti, Susana G. Rodrigues, Federica Perazza, Luigi Colecchia, Giovanni Marasco, Matteo Renzulli, Giovanni Barbara, Francesco Azzaroli, Annalisa Berzigotti, Antonio Colecchia, and Federico Ravaioli

Sarcopenia evaluated by EASL/AASLD computed tomography-based criteria predicts mortality in patients with cirrhosis: A systematic review and meta-analysis

Elton Dajti, Susana G. Rodrigues, Federica Perazza, Luigi Colecchia, Giovanni Marasco, Matteo Renzulli, Giovanni Barbara, Francesco Azzaroli, Annalisa Berzigotti, Antonio Colecchia, Federico Ravaioli

Supplementary Material 1 2
Fig. S1 3
Fig. S2 4
Fig. S3 5
Fig. S4 6
Table S1. 7
Table S2 8

Supplementary Material 1 - Electronic Search strategy.

a) PUBMED (n=2227)

("liver disease*" OR "cirrhosis" OR "cirrhotic" OR ("Liver cirrhosis" [MeSH Terms])) AND ("sarcopenia" OR "sarcopenic" OR "muscle wasting" OR "muscle atrophy" OR "muscle depletion" OR "muscle mass" OR "skeletal muscle*" OR "psoas muscle*" OR ("sarcopenia" [MeSH Terms]))

b) SCOPUS (n=2681)

TITLE-ABS-KEY(("liver disease*" OR "cirrhosis") AND ("sarcopenia" OR "sarcopenic" OR "muscle wasting" OR "muscle depletion" OR "skeletal muscle*"))

c) Embase (n=2686)

'sarcopeni$' AND ('liver cirrhosis'/exp OR 'liver disease'/exp)

d) Cochrane Library (n=144)
('liver disease' OR 'cirrhosis*') AND ('sarcopenia' OR 'sarcopenic')

Fig. S1 – Summary prevalence of sarcopenia in the included studies.

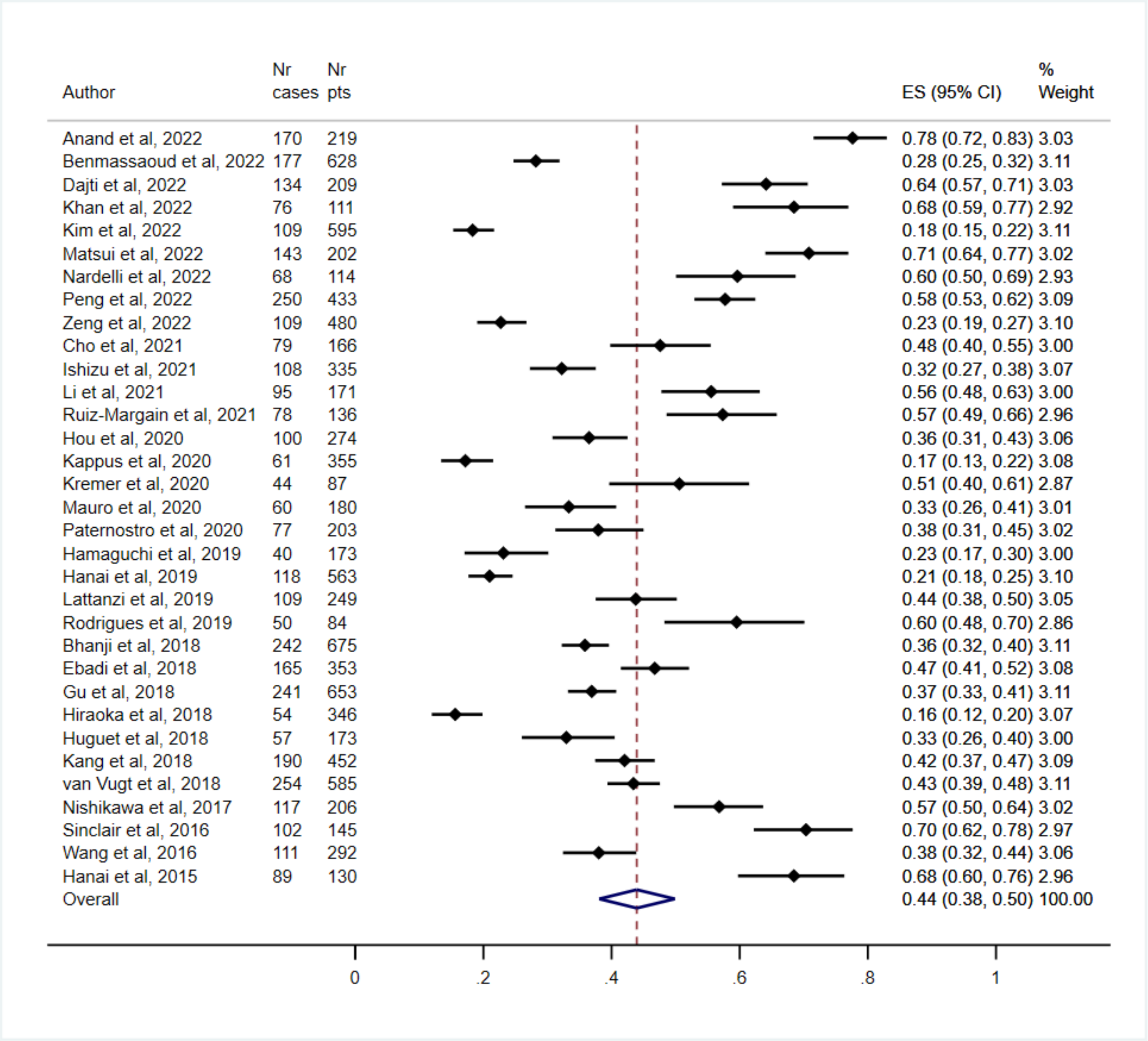

Fig. S2 – Risk of mortality in the presence of sarcopenia in cirrhotic patients.

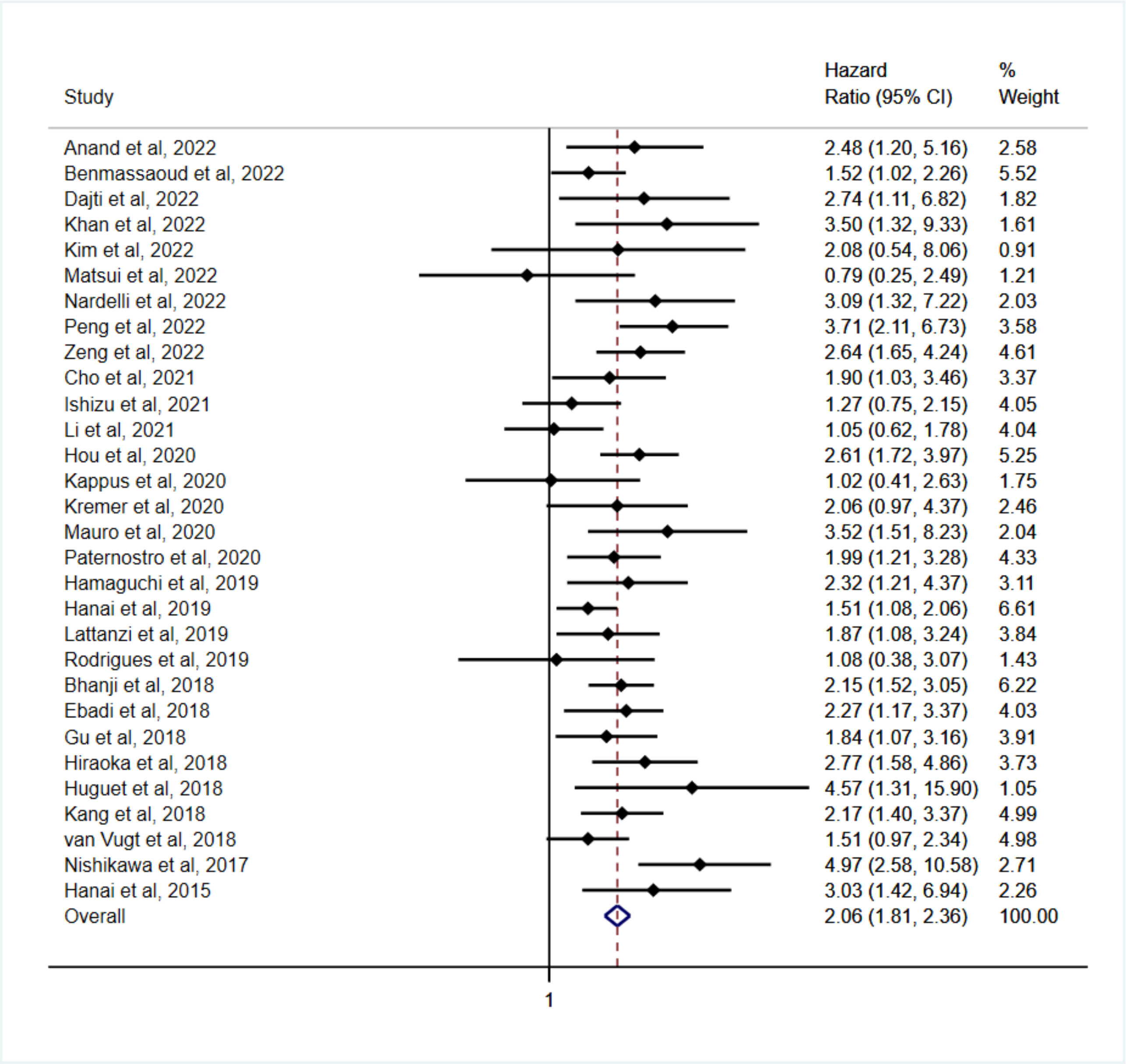

Fig. S3 – Risk of mortality with each unit increase of skeletal muscle index.

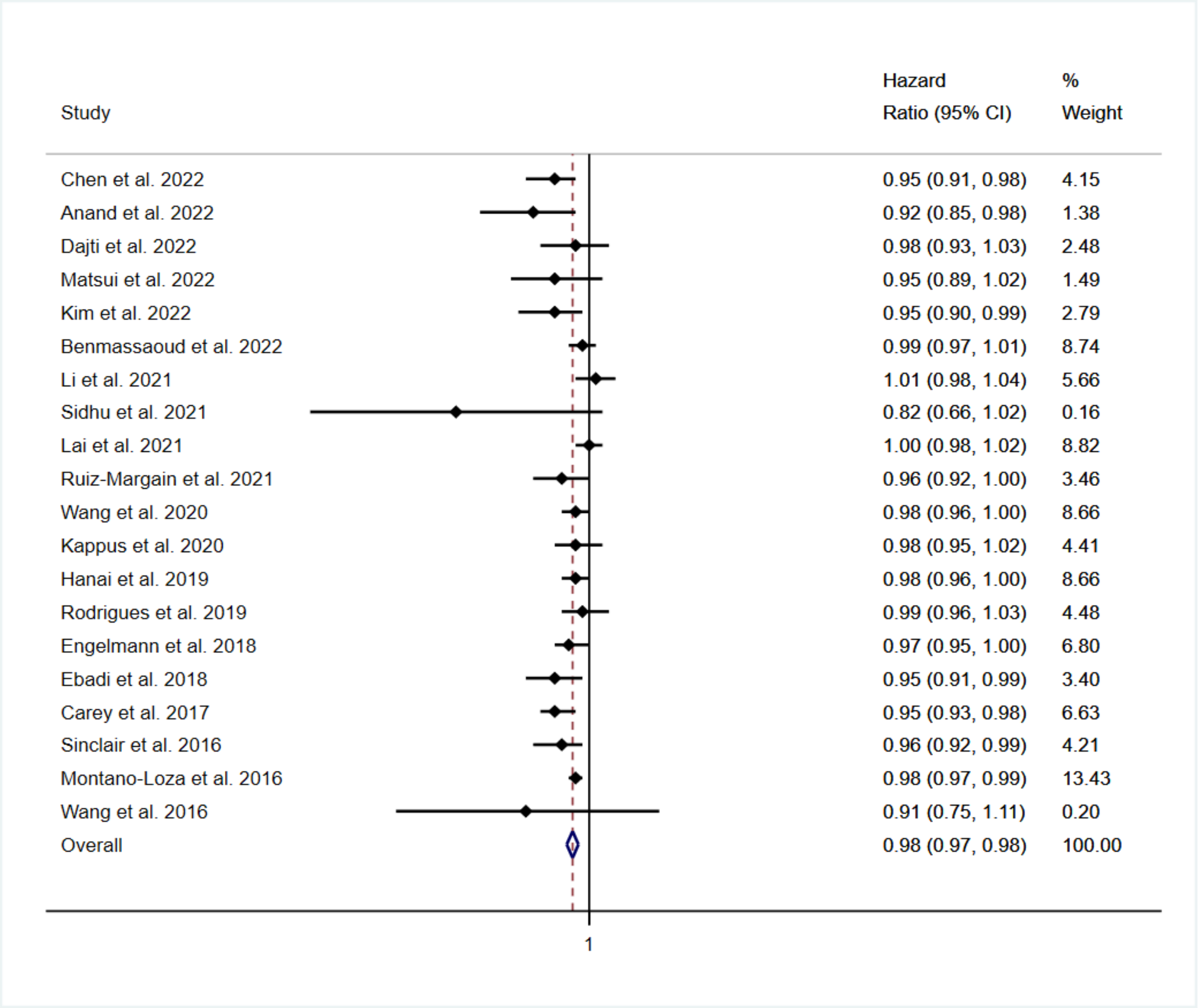

Fig. S4 - Publication bias was using the funnel plot.

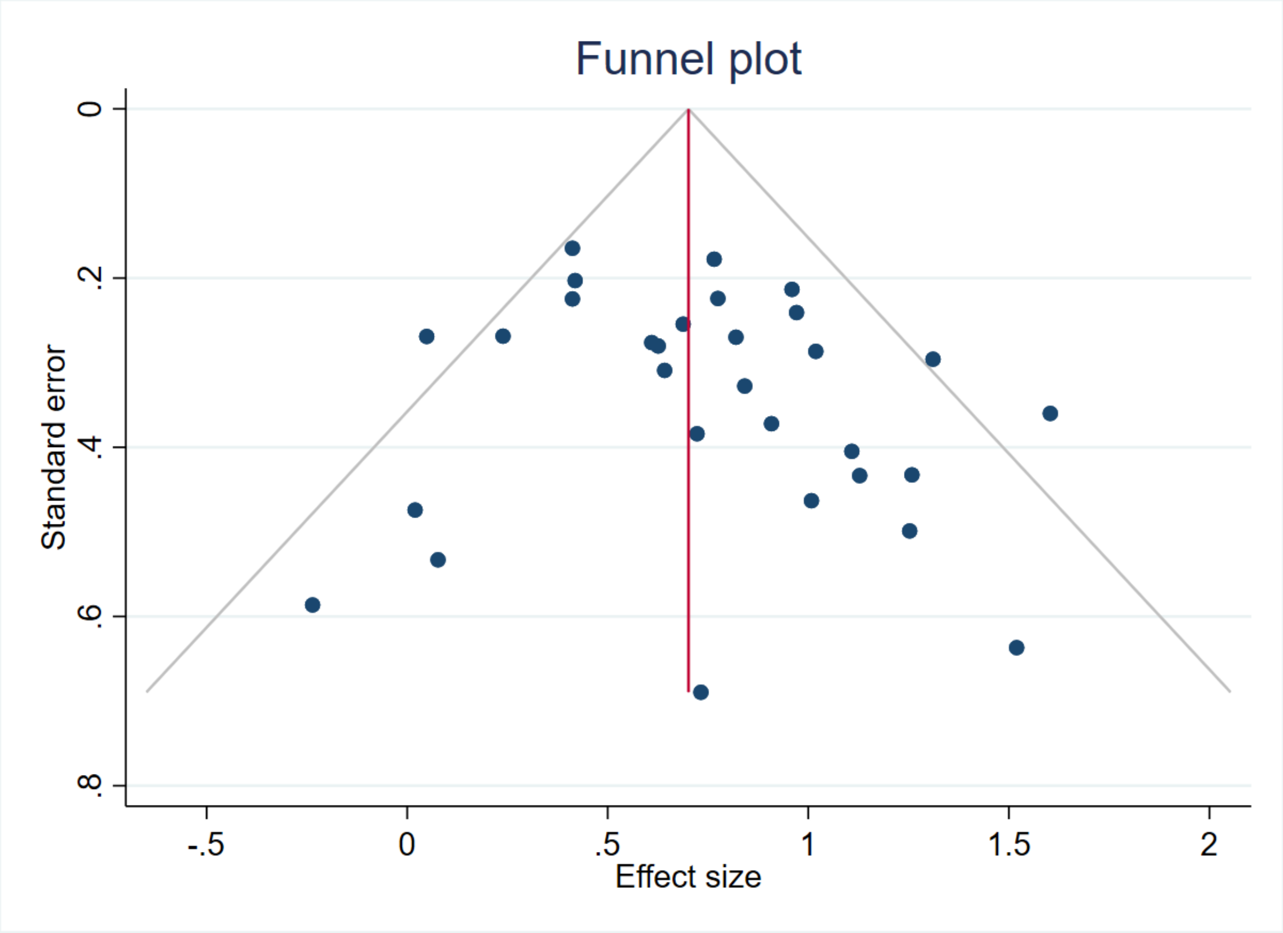

Table S1 – Variables included in the multivariate model in the evaluated studies.

Author, Year	Variables included in the multivariate analysis	Author, Year	Variables included in the multivariate
Anand et al, 2022	N/A	Paternostro et al, 2020	Age, gender, MELD, hepatic venous pressure gradient, albumin
Benmassaoud et al, 2022	N/A	Wang et al, 2020	Age, gender, Child-Pugh
Chen et al, 2022	Age, MELD, hepatic encephalopathy, infection	Hamaguchi et al, 2019	MELD, Neutrophil-to-lymphocyte ratio, myosteatosis, visceral fat
Dajti et al, 2022	Age, gender, clinically significant portal hypertension	Hanai et al, 2019	N/A
Khan et al, 2022	Sepsis, APACHE II score	Lattanzi et al, 2019	MELD, hepatic encephalopathy, myosteatosis
Kim et al, 2022	N/A	Rodrigues et al, 2019	MELD
Matsui et al, 2022	MELD, hepatic venous pressure gradient, changes in SMI	Bhanji et al, 2018	MELD, hepatic encephalopathy, Na, albumin, myosteatosis
Nardelli et al, 2022	Age, MELD, porto-systemic shunts, hepatic encephalopathy	Ebadi et al, 2018	MELD, albumin, hepatocellular carcinoma
Peng et al, 2022	Age, MELD, platelet count, alpha-fetoprotein, complications of cirrhosis	Engelmann et al, 2018	Age, gender, body mass index, MELD
Zeng et al, 2022	N/A	Gu et al, 2018	Etiology, platelet count, INR, bilirubin, albumin, creatinine, Na
Cho et al, 2021	MELD, hepatic vein pressure gradient, bone density	Hiraoka et al, 2018	Bilirubin, albumin, Child-Pugh, hepatocellular carcinoma
Ishizu et al, 2021	Age, gender, etiology, Child-Pugh	Huguet et al, 2018	N/A
Lai et al, 2021	Age, gender, body mass index, MELD, albumin	Kang et al, 2018	Gender, etiology, Child-Pugh, hepatic venous pressure gradient
Li et al, 2021	N/A	van Vugt et al, 2018	Age, MELD, hepatic encephalopathy
Ruiz-Margain et al, 2021	Age, MELD	Nishikawa et al, 2017	Age, body mass index, malnutrition, triglycerides, renal function, hepatocellular carcinoma, biomarkers
Sidhu et al, 2021	Age, gender, MELD	Sinclair et al, 2016	Age, MELD
Hou et al, 2020	Age, MELD, Child-Pugh, myosteatosis, visceral adipose tissue	Wang et al, 2016	Gender, MELD-Na, hepatocellular carcinoma, body mass index
Kappus et al, 2020	MELD	Hanai et al, 2015	Child-Pugh, use of branched chain amino acids
Kremer et al, 2020	MELD, albumin, hepatic encephalopathy, frailty	Durand et al, 2014	MELD
Mauro et al, 2020	MELD, albumin, cystatin-C		

Abbreviations APACHE: Acute Physiology and Chronic Health Evaluation MELD: Model for End-stage Liver Disease; Na: Natrium; N/A: not applicable; SMI: skeletal muscle index.

Table S2 – Prevalence of sarcopenia in subgroup analysis.

Subgroup	Nr. studies	Summary prevalence (95%-CI)	I^2	Meta-regression coefficient	p-value
**Overall**	33	44% (38-50%)	97.2%		
**Study type**				0.35 (-0.31, 0.996)	0.299
Retrospective	26	42% (36-48%)	96.9%		
Prospective	7	51% (34-67%)	98.2%		
**Study size**				-0.96 (-1.54, -0.38)	0.001
<150 pts	7	63% (57-68%)	97.7%		
>150 pts	26	39% (33-45%)	59.6%		
**Region**				-0.04 (-0.37, 0.29)	0.817
Asia	17	44% (34-53%)	98.1%		
Europe	9	46% (38-50%)	93.8%		
North America	5	38% (27-51%)	96.3%		
Other	2	50% (44-55%)	.		
**Etiology**					
Viral	20	40% (33-48%)	97.3%	-0.53 (-1.99, 0.93)	0.475
ArLD	11	51% (41-60%)	95.7%	0.94 (-0.55, 2.44)	0.217
Other	2	45% (39-52%)	.		
**Inclusion of HCC patients**				-1.32 (-2.71, 0.04)	0.056
No	18	48% (40-57%)	97.3%		
Yes	15	39% (31-47%)	97.1%		
**Main inclusion criteria**				0.25 (0.01, 0.49)	0.039
Cirrhosis	19	37% (30-44%)	95.2%		
LT candidates	11	46% (37-55%)	97.7%		
ACLF	3	60% (53-66%)	.		
**Liver function**				0.03 (-0.04, 0.09)	0.416
Mean MELD <15	18	46% (38-53%)	97.1%		
Mean MELD ≥15	10	44% (33-55%)	96.9%		
**Definition**				-0.24 (-1.002, 0.53)	0.548
Skeletal muscle-based	28	43% (36-50%)	97.5%		
Psoas muscle-based	5	38% (22-55%)	96.7%		

Abbreviations: ACLF: acute-on-chronic liver failure; ArLD: alcohol-related liver disease; CI: confidence interval; LT: liver transplant; MELD: Model for End-stage Liver Disease.

Section and Topic	Item #	Checklist item	Location where item is reported
**TITLE**			
Title	1	Identify the report as a systematic review.	1
**ABSTRACT**			
Abstract	2	See the PRISMA 2020 for Abstracts checklist.	3
**INTRODUCTION**			
Rationale	3	Describe the rationale for the review in the context of existing knowledge.	5
Objectives	4	Provide an explicit statement of the objective(s) or question(s) the review addresses.	6
**METHODS**			
Eligibility criteria	5	Specify the inclusion and exclusion criteria for the review and how studies were grouped for the syntheses.	6-7
Information sources	6	Specify all databases, registers, websites, organisations, reference lists and other sources searched or consulted to identify studies. Specify the date when each source was last searched or consulted.	6
Search strategy	7	Present the full search strategies for all databases, registers and websites, including any filters and limits used.	6
Selection process	8	Specify the methods used to decide whether a study met the inclusion criteria of the review, including how many reviewers screened each record and each report retrieved, whether they worked independently, and if applicable, details of automation tools used in the process.	6-7
Data collection process	9	Specify the methods used to collect data from reports, including how many reviewers collected data from each report, whether they worked independently, any processes for obtaining or confirming data from study investigators, and if applicable, details of automation tools used in the process.	7-8
Data items	10a	List and define all outcomes for which data were sought. Specify whether all results that were compatible with each outcome domain in each study were sought (e.g. for all measures, time points, analyses), and if not, the methods used to decide which results to collect.	7-8
	10b	List and define all other variables for which data were sought (e.g. participant and intervention characteristics, funding sources). Describe any assumptions made about any missing or unclear information.	7
Study risk of bias assessment	11	Specify the methods used to assess risk of bias in the included studies, including details of the tool(s) used, how many reviewers assessed each study and whether they worked independently, and if applicable, details of automation tools used in the process.	7
Effect measures	12	Specify for each outcome the effect measure(s) (e.g. risk ratio, mean difference) used in the synthesis or presentation of results.	8
Synthesis methods	13a	Describe the processes used to decide which studies were eligible for each synthesis (e.g. tabulating the study intervention characteristics and comparing against the planned groups for each synthesis (item #5)).	8-9
	13b	Describe any methods required to prepare the data for presentation or synthesis, such as handling of missing summary statistics, or data conversions.	8
	13c	Describe any methods used to tabulate or visually display results of individual studies and syntheses.	8-9
	13d	Describe any methods used to synthesize results and provide a rationale for the choice(s). If meta-analysis was performed, describe the model(s), method(s) to identify the presence and extent of statistical heterogeneity, and software package(s) used.	8-9
	13e	Describe any methods used to explore possible causes of heterogeneity among study results (e.g. subgroup analysis, meta-regression).	8-9
	13f	Describe any sensitivity analyses conducted to assess robustness of the synthesized results.	8-9
Reporting bias assessment	14	Describe any methods used to assess risk of bias due to missing results in a synthesis (arising from reporting biases).	9
Certainty	15	Describe any methods used to assess certainty (or confidence) in the body of evidence for an outcome.	8-9

Section and Topic	Item #	Checklist item	Location where item is reported
assessment			
**RESULTS**			
Study selection	16a	Describe the results of the search and selection process, from the number of records identified in the search to the number of studies included in the review, ideally using a flow diagram.	9
	16b	Cite studies that might appear to meet the inclusion criteria, but which were excluded, and explain why they were excluded.	9
Study characteristics	17	Cite each included study and present its characteristics.	9-10
Risk of bias in studies	18	Present assessments of risk of bias for each included study.	9
Results of individual studies	19	For all outcomes, present, for each study: (a) summary statistics for each group (where appropriate) and (b) an effect estimate and its precision (e.g. confidence/credible interval), ideally using structured tables or plots.	10-12
Results of syntheses	20a	For each synthesis, briefly summarise the characteristics and risk of bias among contributing studies.	12
	20b	Present results of all statistical syntheses conducted. If meta-analysis was done, present for each the summary estimate and its precision (e.g. confidence/credible interval) and measures of statistical heterogeneity. If comparing groups, describe the direction of the effect.	10-12
	20c	Present results of all investigations of possible causes of heterogeneity among study results.	10-12
	20d	Present results of all sensitivity analyses conducted to assess the robustness of the synthesized results.	12
Reporting biases	21	Present assessments of risk of bias due to missing results (arising from reporting biases) for each synthesis assessed.	12
Certainty of evidence	22	Present assessments of certainty (or confidence) in the body of evidence for each outcome assessed.	12
**DISCUSSION**			
Discussion	23a	Provide a general interpretation of the results in the context of other evidence.	13-14
	23b	Discuss any limitations of the evidence included in the review.	13-14
	23c	Discuss any limitations of the review processes used.	13
	23d	Discuss implications of the results for practice, policy, and future research.	15-16
**OTHER INFORMATION**			
Registration and protocol	24a	Provide registration information for the review, including register name and registration number, or state that the review was not registered.	6
	24b	Indicate where the review protocol can be accessed, or state that a protocol was not prepared.	6
	24c	Describe and explain any amendments to information provided at registration or in the protocol.	6
Support	25	Describe sources of financial or non-financial support for the review, and the role of the funders or sponsors in the review.	2
Competing interests	26	Declare any competing interests of review authors.	2
Availability of data, code and other materials	27	Report which of the following are publicly available and where they can be found: template data collection forms; data extracted from included studies; data used for all analyses; analytic code; any other materials used in the review.	2

From: Page MJ, McKenzie JE, Bossuyt PM, Boutron I, Hoffmann TC, Mulrow CD, et al. The PRISMA 2020 statement: an updated guideline for reporting systematic reviews. BMJ 2021;372:n71. doi: 10.1136/bmj.n71

MOOSE (Meta-analyses Of Observational Studies in Epidemiology) Checklist

A reporting checklist for Authors, Editors, and Reviewers of Meta-analyses of Observational Studies. You must report the page number in your manuscript where you consider each of the items listed in this checklist. If you have not included this information, either revise your manuscript accordingly before submitting or note N/A.

Reporting Criteria	Reported (Yes/No)	Reported on Page No.
**Reporting of Background**		
Problem definition	Yes	5
Hypothesis statement	Yes	6
Description of Study Outcome(s)	Yes	6
Type of exposure or intervention used	Yes	5-6
Type of study design used	Yes	5-6
Study population	Yes	5-6
**Reporting of Search Strategy**		
Qualifications of searchers (eg, librarians and investigators)	Yes	6
Search strategy, including time period included in the synthesis and keywords	Yes	6
Effort to include all available studies, including contact with authors	Yes	7
Databases and registries searched	Yes	6
Search software used, name and version, including special features used (eg, explosion)	Yes	6
Use of hand searching (eg, reference lists of obtained articles)	Yes	6
List of citations located and those excluded, including justification	Yes	6, 9
Method for addressing articles published in languages other than English	Yes	6
Method of handling abstracts and unpublished studies	Yes	6
Description of any contact with authors	Yes	7
**Reporting of Methods**		
Description of relevance or appropriateness of studies assembled for assessing the hypothesis to be tested	Yes	6
Rationale for the selection and coding of data (eg, sound clinical principles or convenience)	Yes	6
Documentation of how data were classified and coded (eg, multiple raters, blinding, and interrater reliability)	Yes	6-7
Assessment of confounding (eg, comparability of cases and controls in studies where appropriate	Yes	7-8

Reporting Criteria	Reported (Yes/No)	Reported on Page No.
Assessment of study quality, including blinding of quality assessors; stratification or regression on possible predictors of study results	Yes	7
Assessment of heterogeneity	Yes	8
Description of statistical methods (eg, complete description of fixed or random effects models, justification of whether the chosen models account for predictors of study results, dose-response models, or cumulative meta-analysis) in sufficient detail to be replicated	Yes	8-9
Provision of appropriate tables and graphics	Yes	8-9
**Reporting of Results**		
Table giving descriptive information for each study included	Yes	9
Results of sensitivity testing (eg, subgroup analysis)	Yes	10-12
Indication of statistical uncertainty of findings	Yes	10-12
**Reporting of Discussion**		
Quantitative assessment of bias (eg, publication bias)	Yes	14
Justification for exclusion (eg, exclusion of non–English-language citations)	Yes	13
Assessment of quality of included studies	Yes	14
**Reporting of Conclusions**		
Consideration of alternative explanations for observed results	Yes	14-16
Generalization of the conclusions (ie, appropriate for the data presented and within the domain of the literature review)	Yes	15-16
Guidelines for future research	Yes	15-16
Disclosure of funding source	Yes	2

Once you have completed this checklist, please save a copy and upload it as part of your submission. DO NOT include this checklist as part of the main manuscript document. It must be uploaded as a separate file.
